# The Brassicaceae-restricted gene *TRQA1*, acting opposite to *QQS*, modulates starch and protein accumulation in Arabidopsis

**DOI:** 10.3389/fpls.2026.1862971

**Published:** 2026-07-07

**Authors:** Rezwan Tanvir, Caroline Kercheval, Kelsi White, Emma Koeppen, Mary Virginia Miller, Sharnali Das, Lei Wang, Ling Li

**Affiliations:** 1 Department of Biological Sciences, Mississippi State University, Mississippi State, MS, United States; 2 College of Life Sciences, Shihezi University, Shihezi, China

**Keywords:** carbon partitioning, iron homeostasis, MYB103, NF-YC4, QQS, taxonomically restricted genes

## Abstract

**Introduction:**

The *Arabidopsis thaliana* orphan gene Qua-Quine Starch (*QQS*) increases leaf and seed protein content and reduces starch accumulation across multiple plant species without compromising yield. Through microarray analysis, we found that suppression of *QQS* expression was associated with marked upregulation of Taxonomically Restricted *QQS* Associated 1 (*TRQA1*), a Brassicaceae-restricted gene. Although *TRQA1* has previously been implicated in iron accumulation and homeostasis in Arabidopsis, its role in plant metabolism has not been examined.

**Methods:**

Here, we used Arabidopsis lines with altered *TRQA1* expression, including overexpression, RNAi knockdown, and T-DNA insertion lines, together with *TRQA1* promoter-reporter constructs, to characterize its expression pattern and metabolic function.

**Results:**

*TRQA1* was broadly expressed across organs and developmental stages, with strong expression in cotyledons, radicles, vascular tissues, young leaves, the shoot meristem, and root branching points. Subcellular localization analysis detected *TRQA1* in chloroplasts, the plasma membrane, and the cytosol. Functionally, *TRQA1* knockdown or T-DNA insertion increased total protein content by 8–15% in leaves and 11–27% in seeds and reduced leaf starch accumulation by 13–34% in leaves (*P* < 0.05), without detectable penalties on growth or seed yield. In contrast, *TRQA1* overexpression increased leaf starch by 16–25% while reducing leaf protein by 8–21% and seed protein by 7–9% (*P* < 0.05 for all comparisons), and additionally reduced plant biomass by 19% and total seed yield by 14%. AlphaFold2-Multimer modeling suggested a putative interaction between TRQA1 and MYB103, which may, in turn, associate with NF-YC4, providing a hypothesis for an indirect link between TRQA1 and the QQS-NF-YC4 regulatory module.

**Discussion:**

Together, these findings identify TRQA1 as a Brassicaceae-restricted regulator of carbon and nitrogen partitioning that controls starch and protein content in Arabidopsis, expanding our understanding of how lineage-specific genes contribute to metabolic regulation.

## Introduction

Taxonomically restricted genes (TRGs), also referred to as lineage-specific genes, are genes confined to a particular clade or taxon and lacking recognizable homologs, conserved motifs, or clear ancestral relationships outside that lineage (Arendsee et al., 2014; Johnson, 2018). Orphan genes (OGs) represent the most restricted subset of TRGs, being found in only a single species (Arendsee et al., 2014; Johnson, 2018). TRGs are estimated to constitute approximately 10-20% of the plant genome and are increasingly recognized as important contributors to phenotypic innovation. They have been implicated in diverse biological processes, including plant growth and development, metabolism, hormone signaling, stress responses, defense, and other lineage-specific traits that may confer adaptive advantages (Arendsee et al., 2014; Johnson, 2018). Despite their prevalence and apparent functional importance, TRGs and OGs remain poorly characterized, largely because the absence of homologs limits computational inference and necessitates extensive experimental validation. Elucidating the functions of these genes is therefore essential for understanding how evolutionary novelty arises and how lineage-specific genes contribute to biological diversity.

The orphan gene Qua-Quine Starch (*QQS*; AT3G30720) provides a compelling example of the biological significance of OGs. *QQS* is unique to *Arabidopsis thaliana* and functions as a regulator of carbon and nitrogen allocation. Transgenic expression of *QQS* in multiple crop species has been shown to increase protein content, reduce starch accumulation, and enhance agronomic performance without measurable penalties on growth or yield (Li et al., 2009; Li and Wurtele, 2015; Li et al., 2015; O’conner et al., 2018; Qi et al., 2019; O’conner et al., 2021; Tanvir et al., 2022a, b; Wang et al., 2023a). Mechanistically, QQS interacts with NF-YC4, a component of the conserved NF-Y transcription factor complex, thereby linking a species-specific orphan gene to a deeply conserved regulatory module (Li et al., 2015; Qi et al., 2019; Wang et al., 2023b). This finding highlights how lineage-specific genes can acquire important regulatory roles through association with conserved cellular machinery.

**Figure 1:**
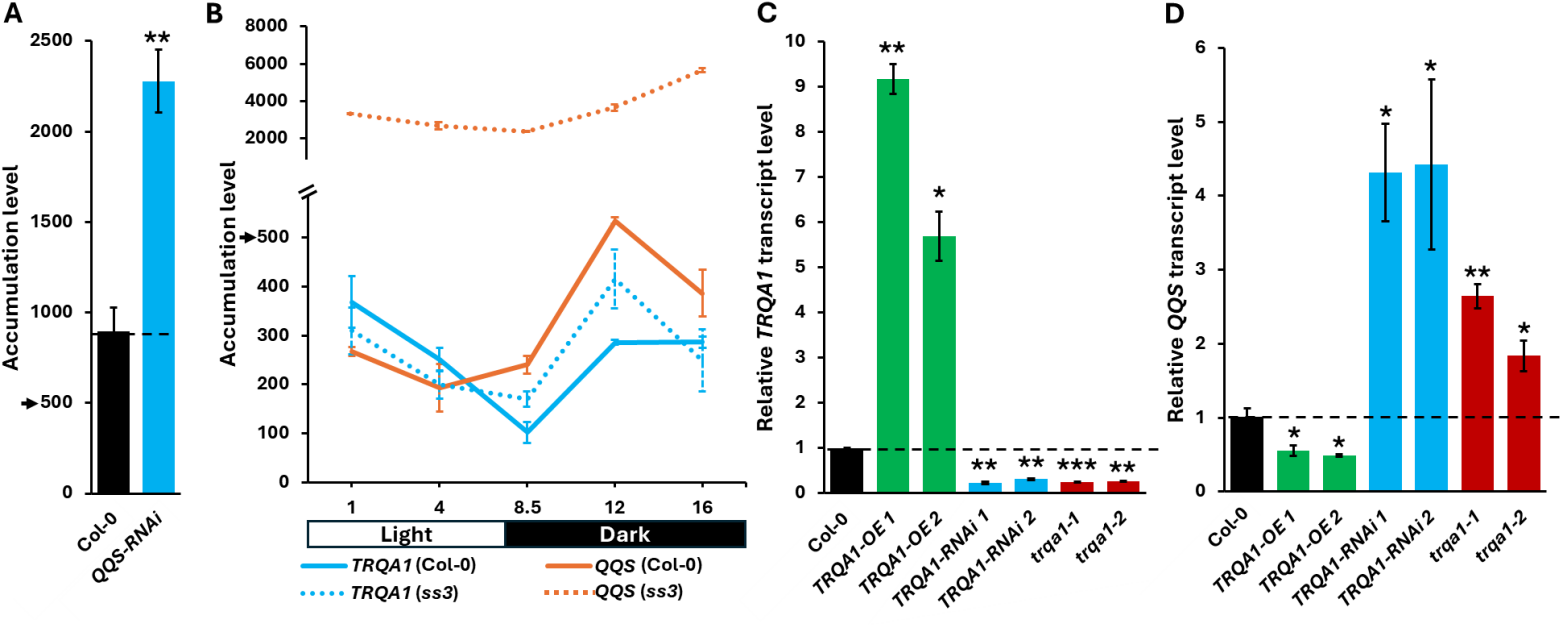
Reciprocal expression patterns of *TRQA1* and *QQS* in mutant and transgenic lines. **(A)** *TRQA1* signal intensity values in *QQS-RNAi* under long-day (LD) conditions, obtained from the microarray dataset of Li et al (Li et al., 2009). **(B)** Microarray data for Col-0 and *ss3* under short-day (SD) conditions, obtained from (Li et al., 2009). The mean transcript accumulation level for each chip was normalized to 500 (indicated by arrows). **(C)** Relative transcript levels of *TRQA1* in leaf tissue of transgenic Arabidopsis plants, quantified by RT-qPCR using *ACT2* as the reference gene. **(D)** Relative transcript levels of *QQS* in leaf tissue of transgenic Arabidopsis plants, quantified by RT-qPCR using *ACT2* as the reference gene. All data are presented as mean ± standard error of the mean (SEM) (*n* = 3). Differences between mutant or transgenic lines and their corresponding WT controls were determined by Student’s *t*-test: ***P< 0.001; **P< 0.01; *P< 0.05.

Taxonomically Restricted *QQS* Associated 1 (*TRQA1*; AT1G47400) is a Brassicaceae-restricted TRG that emerged as a candidate regulator in our analysis of *QQS*-associated gene expression. Preliminary data showed that *TRQA1* is strongly upregulated when *QQS* is downregulated (**Figure 1A**), suggesting a functional relationship between these two lineage-restricted genes and raising the possibility that *TRQA1* may also participate in the regulation of carbon and nitrogen allocation. Sequence analysis further confirmed that *TRQA1* is highly conserved within Brassicaceae but absent outside this family, consistent with its classification as a TRG (**Supplementary Figure 1**). Although *TRQA1*, also known as FE-UPTAKE-INDUCING PEPTIDE 3 (*FEP3*) (Hirayama et al., 2018) and IRON MAN 1 (*IMA1*) (Grillet et al., 2018), has been associated with iron accumulation and homeostasis through activation of iron deficiency-responsive genes, its potential role in primary metabolism has not been investigated. In particular, whether *TRQA1* influences carbon and nitrogen partitioning, and thereby affects starch and protein accumulation, remains unknown.

Iron serves as an essential cofactor for key enzymes in both photosynthetic electron transport and nitrogen assimilation, including ferredoxin, nitrite reductase, and glutamate synthase (GOGAT), all of which depend on iron-sulfur clusters or heme-iron for activity (Nasar et al., 2022; Yang et al., 2023; Ali et al., 2025; Haque et al., 2026). Because *TRQA1*/*FEP3*/*IMA1* is a phloem peptide that regulates systemic iron distribution (Grillet et al., 2018; Hirayama et al., 2018; Cao et al., 2024), it is plausible that the carbon and nitrogen partitioning phenotypes described here are at least partially downstream of altered iron availability in photosynthetic tissues. This possibility, however, remains unresolved and requires direct experimental investigation.

Here, we investigated the role of *TRQA1* in plant metabolism using a combination of genetic, physiological, and cell biological approaches in *A. thaliana*. We generated and analyzed lines with altered *TRQA1* expression, including overexpression, RNAi knockdown, and T-DNA insertion genotypes, and complemented these analyses with promoter-reporter assays, subcellular localization studies, promoter *cis*-element analysis, and protein interaction prediction. We hypothesized that *TRQA1* modulates starch and protein accumulation through a regulatory module that overlaps with or antagonizes the QQS-NF-YC4 pathway, and that its expression domain and potential interaction partners can be inferred from genetic and computational evidence. Our results identify *TRQA1* as a previously uncharacterized regulator of carbon and nitrogen partitioning that modulates starch and protein accumulation in Arabidopsis. These findings expand the functional landscape of taxonomically restricted genes and support the view that lineage-specific genes can play central roles in conserved metabolic processes, although the translational relevance of *TRQA1* for crop improvement will depend on future resolution of the starch-protein trade-off and the yield consequences associated with altered *TRQA1* expression.

## Materials and methods

### Plant material

For overexpression, the Arabidopsis *TRQA1* coding sequence (AT1G47400 CDS; see **Supplementary Table 1**) was placed under the control of the cauliflower mosaic virus (CaMV) 35S promoter in the pB2GW7 vector, as described previously (Li and Wurtele, 2015).

For the GUS construct, the *TRQA1* promoter was fused to the GUS/GFP reporter gene in the pBGWFS7 binary vector (Karimi et al., 2002). For the GFP construct, the *TRQA1* promoter and the coding sequence encoding the full-length TRQA1 protein were fused to the GUS/GFP reporter gene in pBGWFS7 (Karimi et al., 2002), so that any intrinsic targeting signal in TRQA1 would be expected to direct the fusion protein to the same subcellular location as the native protein.

*TRQA1*-*RNAi* knockdown lines were generated by transformation with a construct expressing a sense fragment of the *TRQA1* coding sequence in the pB7GWIWG2(II) vector for hairpin RNA expression (Karimi et al., 2002). All constructs were generated using the Gateway cloning system (Life Technologies) as previously described (Li et al., 2007; Li and Wurtele, 2015). Transgenic Arabidopsis plants were produced by *Agrobacterium*-mediated transformation.

Additionally, the T-DNA insertion lines SALK_130118 (*trqa1-1*) and GK-961C11 (*trqa1-2*) were obtained from the Arabidopsis Biological Resource Center (ABRC, https://abrc.osu.edu/) and the Nottingham Arabidopsis Stock Centre (NASC, https://arabidopsis.info/), respectively. Residual *TRQA1* transcript detected in these lines by RT-qPCR is consistent with amplification of truncated transcripts upstream of the T-DNA insertion site, a well-documented phenomenon in confirmed insertion mutants (Wang, 2008; Kadam et al., 2014).

To minimize the influence of positional insertion effects and transgene silencing, more than 20 independent transformants were initially generated and screened in the T_2_ generation by RT-qPCR for transgene expression levels. From this pool, lines showing the highest and most consistent overexpression or knockdown of *TRQA1* relative to the wild type (WT) were selected for phenotypic characterization. Screening a larger pool of primary transformants and selecting lines on the basis of transgene expression prior to phenotypic analysis is a common practice in plant functional genomics and is used to reduce variability associated with positional effects on transgene expression. Although zygosity was not directly confirmed beyond PCR-based detection of the transgene, two independent lines per transgenic class were included to reduce the likelihood of insertion-specific artifacts, and the consistency of phenotypes across both lines supports the robustness of the conclusions.

### Growing conditions

Transgenic Arabidopsis plants were grown in a growth chamber under long-day conditions (16 h light/8 h dark) at a light intensity of approximately 132 μmol photons·m^-2^·s^-1^ PAR, provided by cool white fluorescent lamps (Philips F25T8/TL841, 25W, 4100K color temperature), at 22 °C and 60% relative humidity. Individual plants were grown one per pot in pots measuring 6 cm x 6 cm x 5.5 cm, filled completely with Miracle-Gro Garden Soil (Vegetable & Herbs; N-P-K 0.09-0.05-0.07; pH 5.5-7.5). Pots were initially bottom-watered by soaking to ensure uniform soil saturation at transplanting. Thereafter, water was applied evenly to the soil surface every 2 days throughout the experiment.

For some experiments, including starch analysis and reporter assays using GUS and GFP, plants were first germinated on petri dishes containing 0.5X Murashige and Skoog medium supplemented with 1% sucrose, 0.05% MES, and 0.1% B5 vitamins (1000X stock) under the same growth conditions described above. Seedlings were then transferred to soil when required and grown under the same chamber conditions.

### BLAST and multiple sequence alignment

Proteins with sequence similarity to TRQA1 were identified using NCBI Protein BLAST (BLASTp) (Altschul et al., 1997). Sequence alignments were performed using the NCBI Multiple Sequence Alignment Viewer (version 1.25.0) to visualize conserved regions, and a phylogenetic distance tree was generated using NCBI Tree Viewer.

### TF-binding sites and *cis*-acting DNA element in the *TRQA1* promoter

Putative transcription factor (TF)-binding sites in the *TRQA1* promoter were identified using PlantRegMap (http://plantregmap.gao-lab.org/binding_site_prediction.php (Tian et al., 2019), with the 2-kb region upstream of the *TRQA1* start codon used as the query sequence and a threshold of *P* ≤ 1 × 10^-4^).

*Cis*-acting DNA elements in the same promoter region were identified using PlantCARE (http://bioinformatics.psb.ugent.be/webtools/plantcare/html/; Lescot et al., 2002) and PLACE (https://www.dna.affrc.go.jp/PLACE/?action=newplace; Higo et al., 1999).

### Protein-protein interaction and functional network prediction

The secondary structure of TRQA1 was predicted using AlphaFold (https://alphafold.ebi.ac.uk/entry/Q3ECW0, accessed 8 April 2024; Jumper et al., 2021; Varadi et al., 2021) and I-TASSER (Roy et al., 2010). The predicted secondary structure image from I-TASSER was visualized using PyMol (The PyMOL Molecular Graphics System, Version 3.0; Schrödinger, LLC).

Predicted protein complex structures were generated using AlphaFold2-Multimer (Jumper et al., 2021; Evans et al., 2022) through ColabFold, a Google Colaboratory-hosted implementation that enables accelerated protein complex prediction. ChimeraX (Meng et al., 2023) was used to run AlphaFold2-Multimer predictions in Google Colab and to retrieve the top-ranked predicted complexes using the default parameters. Structures showing putative interactions within 5 Å were generated and exported using ChimeraX.

To further explore functional partners and interaction networks associated with TRQA1, protein–protein association analysis was also performed using the STRING Database (https://string-db.org/, accessed on 10 April 2024; Szklarczyk et al., 2020).

### RT-qPCR, microarray, and RNA-Seq analysis

Total RNA extracted from Arabidopsis leaves was reverse-transcribed into cDNA using M-MuLV reverse transcriptase (New England Biolabs, Ipswich, MA, USA). cDNA concentration was measured using a NanoDrop spectrophotometer (Thermo Fisher Scientific Inc., Waltham, MA, USA), and equal amounts of cDNA were used for RT-qPCR on an Applied Biosystems platform (Applied Biosystems, Waltham, MA, USA).

The following primers were used for *TRQA1*: forward, 5′-TTTGACCATGCTTCCACCGT-3′; reverse, 5′-TCACGCAGCAGGAGCATAAT-3′. ACTIN 2 (*ACT2*) gene (AT3G18780) was used as the internal reference, with the following primers: forward, 5’-GGTAACATTGTGCTCAGTGGTGG-3′; reverse, 5′-AACGACCTTAATCTTCATGCTGC-3’ (Li et al., 2019). Relative transcript abundance was calculated using the 2^−ΔΔCt^ method (Qi et al., 2019).

The *ss3* microarray data were obtained from (Li et al., 2009).

### Starch staining and quantification

Arabidopsis leaves were harvested from intact plants at the end of the light period (16 h light). Leaf tissues were boiled in 80% ethanol for 30 min and then stained with Lugol’s iodine solution (I_2_/KI) for 15 min, followed by destaining in water as previously described (Li et al., 2007). Darker staining was interpreted as indicating higher starch accumulation.

Starch was quantified using the Megazyme D-Glucose Assay Kit (GOPOD format) as previously described (Li et al., 2009). Leaves were collected at the end of the light period (EoL), when starch accumulation is maximal, to maximize sensitivity for detecting genotypic differences. They were boiled in 80% ethanol, ground with a mortar and pestle, washed again with 80% ethanol, and then boiled in sterile water. Starch was enzymatically digested to glucose using α-amylase and amyloglucosidase, and the starch content was calculated from the measured glucose concentration using the appropriate conversion factor.

### Protein quantification and SDS-PAGE

Total protein content in leaves and seeds was determined using a modified Lowry assay (Lowry et al., 1951), as described previously (O’conner et al., 2021). Tissues were ground in liquid nitrogen and homogenized in extraction buffer containing the protease inhibitors 4-aminobenzoic acid and phenylmethylsulfonyl fluoride (PMSF) to minimize protein degradation. Protein concentration was measured colorimetrically using the Pierce™ Modified Lowry Protein Assay Kit (Thermo Fisher Scientific Inc., Waltham, MA, USA).

For Sodium Dodecyl Sulfate–Polyacrylamide Gel Electrophoresis (SDS-PAGE), equal amounts of total protein extracted from Arabidopsis leaves were separated on 10% SDS-PAGE polyacrylamide gels and visualized by staining with Coomassie Brilliant Blue R-250.

### Histochemical analysis using the GUS reporter system

At least 10 independent *TRQA1* promoter::*GUS* lines were initially screened by GUS staining. For detailed analysis, at least three representative independent plants showing similar staining patterns were harvested at each developmental stage and stained according to a previously described protocol (Li et al., 2007).

### Confocal imaging of GFP in transgenic plants

Leaves and roots from at least five independent *TRQA1* promoter::*TRQA1* coding sequence::*GFP* lines were examined by confocal microscopy. Green fluorescence and autofluorescence were observed using a Leica SPE confocal microscope (Leica Microsystems, Wetzlar, Germany) in the Department of Biological Sciences, Mississippi State University, Mississippi State, MS, USA.

### Chlorophyll content measurement

Rosettes from 3-week-old plants were weighed and homogenized in liquid nitrogen using a pestle and mortar. The resulting tissue powder was extracted in 95% ethanol. After centrifugation, the chlorophyll-containing supernatant was collected and kept on ice in the dark to minimize pigment degradation. Absorbance was measured at 664 nm and 649 nm as described by (Lichtenthaler and Buschmann, 2001). Chlorophyll a and chlorophyll b concentrations were calculated using the following equations:


$$\text{Chlorophyll a }\left(µ\text{g}/\text{mL}\right)=13.36\times {\text{A}}_{664}–5.19\times {\text{A}}_{649}$$

$$\text{Chlorophyll b }\left(µ\text{g}/\text{mL}\right)=27.43\times {\text{A}}_{649}–8.12\times {\text{A}}_{664}$$

Total chlorophyll content was calculated as the sum of chlorophyll a and chlorophyll b.

### Statistical analysis

All quantitative data are presented as mean ± standard error of the mean (SEM). Statistical comparisons between each transgenic or mutant line and its corresponding WT control were performed using a two-tailed Student’s *t*-test. Differences were considered statistically significant at *P*< 0.05. The number of biological replicates (*n*) for each experiment is indicated in the corresponding figure legends; *n* refers to independently grown plants or independently harvested samples.

## Results

### *TRQA1* displays an expression pattern opposite to that of *QQS*

Analysis of microarray data from *QQS-RNAi* and Col-0 plants sampled 12 h into the light period under long-day conditions (LD, 16 h light/8 h dark) (Li et al., 2009) showed that *TRQA1* expression increased significantly when *QQS* expression was reduced (**Figure 1A**).

Further evidence for an inverse relationship between *TRQA1* and *QQS* was provided by microarray analysis of the Arabidopsis starch synthase III knockout mutant (*ss3*), a genotype associated with elevated *QQS* expression, compared with Col-0 (Li et al., 2009). Under short-day conditions (SD, 8 h light/16 h dark), *TRQA1* expression in Col-0 was higher than *QQS* expression at the beginning of the light period. This relationship reversed at the onset of darkness and persisted until the end of the dark period (**Figure 1B**). In the *ss3* mutant, *TRQA1* expression was lower than in Col-0 during the light period, but increased at the beginning of the dark period and remained elevated until just before the next light period (**Figure 1B**).

Together, these expression analyses indicate that *TRQA1* exhibits a transcriptional pattern opposite to that of *QQS*, supporting a potential functional relationship between these two genes. Sequence analysis further showed that TRQA1 is highly conserved within the Brassicaceae family but absent outside this lineage, consistent with its classification as a taxonomically restricted gene (**Supplementary Figure 1**; **Supplementary Table 2**). A maximum-likelihood phylogenetic tree of TRQA1 proteins from Brassicaceae species is shown in **Supplementary Figure 2**.

RT-qPCR analysis of the transgenic and mutant lines further showed that *QQS* transcript abundance varied inversely with *TRQA1* expression, with reduced *QQS* transcript levels in *TRQA1-OE* lines and increased levels in *TRQA1-RNAi* and *trqa1* T-DNA insertion lines (**Figure 1D**). Together with the microarray analyses, these results support a reciprocal relationship between *TRQA1* and *QQS*.

To determine whether this inverse expression relationship reflects a functional role in metabolism, we next examined the effects of altered *TRQA1* expression on starch and protein accumulation.

### *TRQA1* alters carbon and nitrogen allocation by increasing starch and decreasing protein accumulation

**Figure 2:**
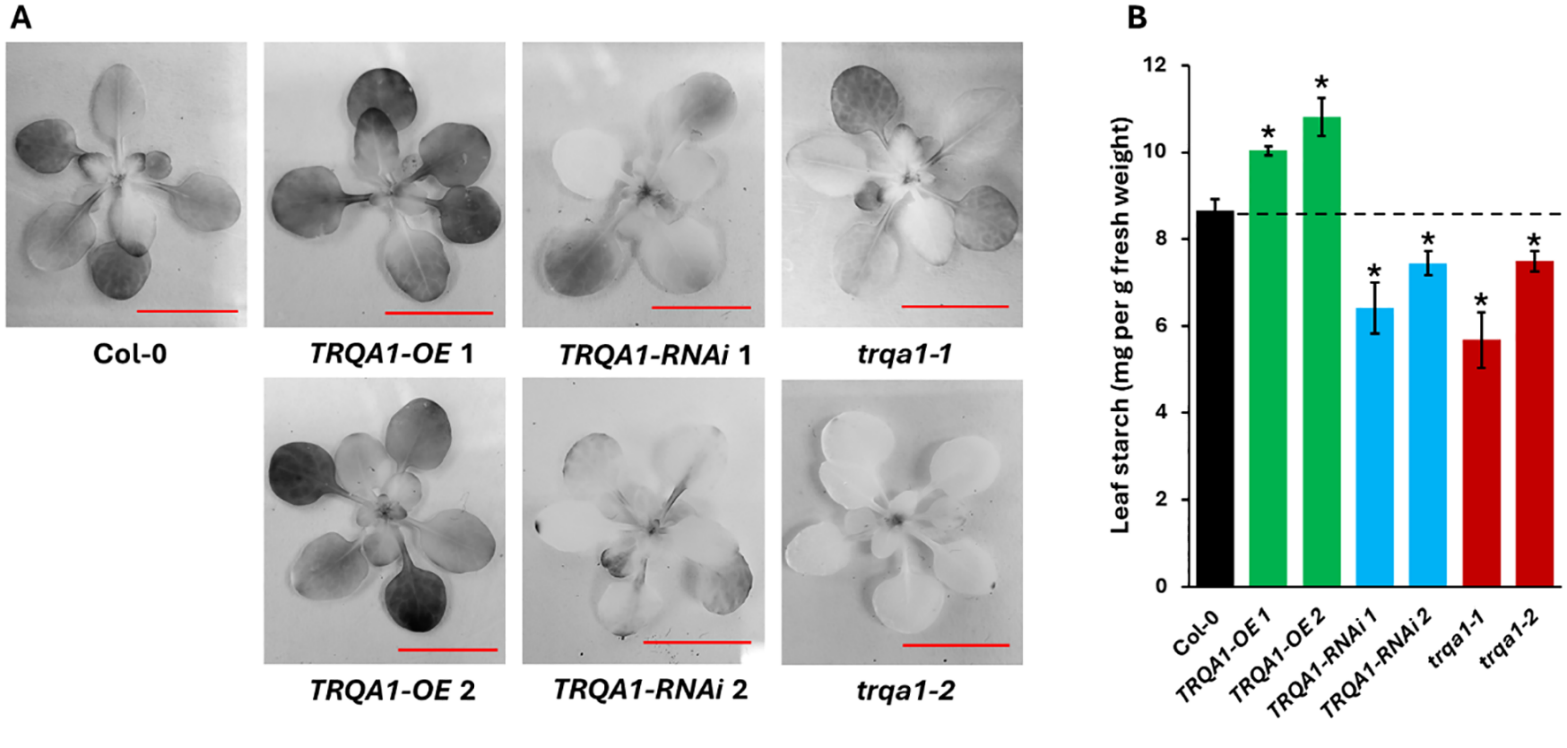
Leaf starch content in *TRQA1* transgenic and mutant lines. **(A)** Iodine staining of leaves from *TRQA1-OE*, *TRQA1-RNAi*, and *trqa1* plants, using I_2_/KI solution. Darker staining indicates higher starch accumulation. **(B)** Leaf starch content in *TRQA1-OE*, *TRQA1-RNAi*, and *trqa1* plants, quantified using Megazyme’s GOPOD starch assay. All plants were grown under LD conditions, and leaves were harvested at the end of the light period. Bar graph data are presented as mean ± SEM (*n* ≥ 3). Differences between *TRQA1-OE*, *TRQA1-RNAi*, or *trqa1* lines and the corresponding WT control were determined using Student’s *t*-test: *P< 0.05. Scale bars in **(A)** = 1 inch.

Expression of *QQS* has been shown to reduce starch accumulation in the leaves, seeds, and tubers across multiple plant species (Li et al., 2009; Li and Wurtele, 2015; Li et al., 2015; O’conner et al., 2018, 2021; Tanvir et al., 2022a, b; Wang et al., 2023a). In contrast, iodine staining showed that *TRQA1* overexpression increased starch accumulation in Arabidopsis leaves at the end of the photoperiod under LD conditions (**Figure 2A**), whereas suppression or T-DNA insertion reduced starch levels.

This pattern was confirmed by quantitative starch measurements (**Figure 2B**). *TRQA1-OE* lines showed a 16–25% increase in leaf starch content, whereas *TRQA1-RNAi* lines showed a 14–26% decrease, and *trqa1* mutant lines showed a 13–34% decrease relative to WT (*P*< 0.05). In both the iodine staining and quantitative assays, *TRQA1-RNAi* and *trqa1* lines showed similar reductions in starch content, with no obvious differences between them. Together, these results indicate that *TRQA1* positively regulates starch accumulation in Arabidopsis leaves.

**Figure 3:**
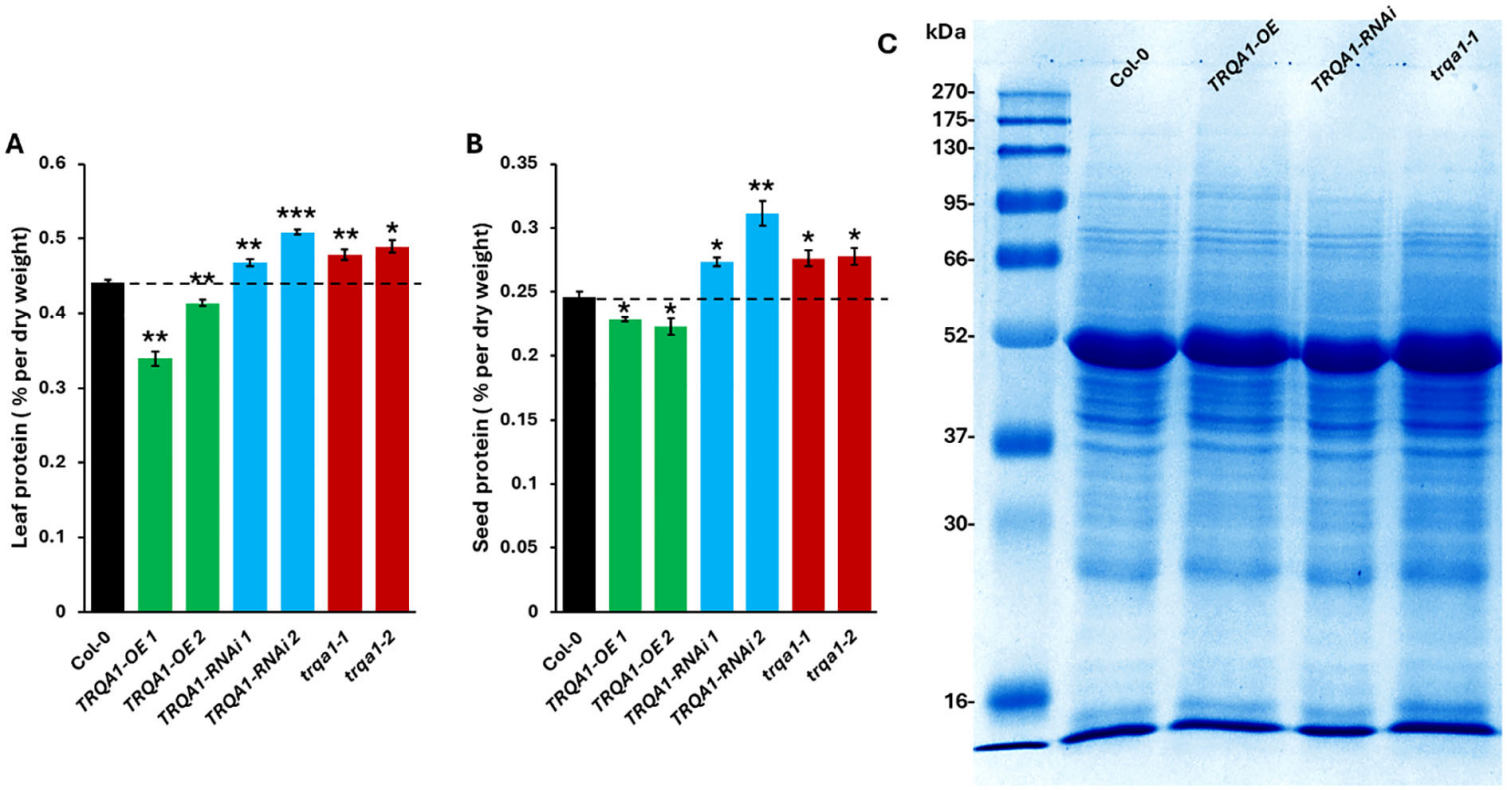
Total protein content in leaves and seeds of *TRQA1* transgenic and mutant lines. The total protein content of leaves **(A)** and seeds **(B)** from *TRQA1-OE*, *TRQA1-RNAi*, and *trqa1* plants, measured using a modified Lowry protein assay. **(C)** SDS-PAGE analysis of total leaf protein from *TRQA1* transgenic and mutant lines. Equal amounts of total protein extracted from Arabidopsis leaves were loaded for electrophoretic separation. A prestained molecular weight marker (16–270 kDa) was included in the leftmost lane of each gel. The experiment was repeated five times using independent biological samples with consistent results; the image shown is representative. All plants were grown under LD conditions. Bar graph data are presented as mean ± SEM (*n* ≥ 3). Differences between *TRQA1-OE*, *TRQA1-RNAi*, or *trqa1* lines and the corresponding WT control were determined using Student’s *t*-test: ***P< 0.001; **P< 0.01; *P< 0.05.

Because starch accumulation is closely linked to carbon and nitrogen partitioning, we next quantified total protein content in leaves and seeds. In contrast to *QQS*, which promotes protein accumulation, *TRQA1* overexpression was associated with reduced protein levels in both leaves and seeds. Compared with Col-0, *TRQA1-OE* plants showed an 8–21% decrease in leaf protein content and a 7–9% decrease in seed protein content (*P*< 0.05; **Figures 3A, B**). Conversely, *TRQA1-RNAi* lines showed an 8–15% increase in leaf protein and an 11–27% increase in seed protein, whereas *trqa1* T-DNA insertion lines showed an 8–12% increase in leaf protein and a 12–13% increase in seed protein relative to Col-0 (*P*< 0.05; **Figures 3A, B**).

To assess whether this increase reflected the accumulation of specific proteins, total leaf protein was analyzed by SDS-PAGE. *TRQA1-RNAi* and *trqa1–1* plants showed no obvious differences in banding patterns relative to WT, suggesting that the increase in protein accumulation was broadly distributed rather than restricted to specific proteins or protein classes (**Figure 3C**). This observation is consistent with our previous findings in *QQS*-expression soybean seeds, in which total protein content increased without detectable changes in the relative proportions of major amino acids (Li and Wurtele, 2015).

Taken together, these findings show that *TRQA1* promotes starch accumulation while reducing total protein content, supporting a role for *TRQA1* in carbon and nitrogen allocation in Arabidopsis. We therefore next asked whether these changes in metabolic composition were accompanied by broader effects on plant growth and performance.

### *TRQA1* overexpression reduces plant growth and seed yield but increases chlorophyll content

**Figure 4:**
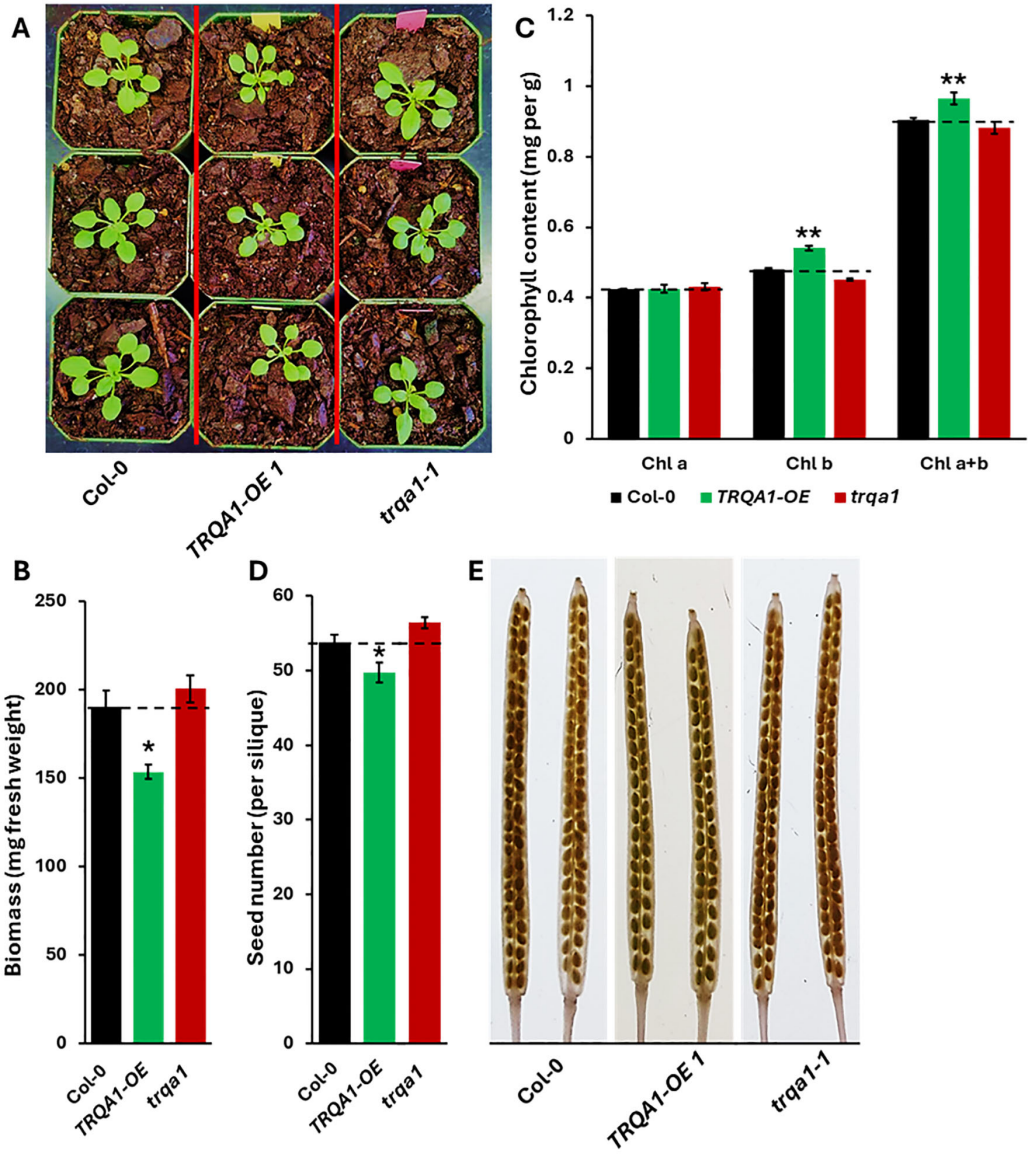
*TRQA1* affects biomass, seed number per silique, and chlorophyll content. **(A)** Morphology of 2-week-old Col-0, *TRQA1-OE 1*, and *trqa1–1* plants. **(B)** Fresh weight (FW) of 3-week-old Col-0, *TRQA1-OE* (pooled mean of *TRQA1-OE 1* and *TRQA1-OE 2*), and *trqa1* (pooled mean of *trqa1–1* and *trqa1-2*) plants (*n* ≥ 8). **(C)** Chlorophyll a, chlorophyll b, and total chlorophyll (a+b) contents in 3-week-old Col-0, *TRQA1-OE* (pooled mean of *TRQA1-OE 1* and *TRQA1-OE 2*), and *trqa1* (pooled mean of *trqa1–1* and *trqa1-2*) plants (*n* ≥ 8). Chlorophyll was extracted with 95% ethanol, and absorbance was measured at 649 and 664 nm for chlorophyll (Chl) quantification. **(D, E)** Seed number per silique in 5-week-old Col-0, *TRQA1-OE* [mean of *TRQA1-OE 1* and *TRQA1-OE 2* in **(D)**; *TRQA1-OE 1* shown in **(E)**], and *trqa1–1* [mean of *trqa1–1* and *trqa1–2* in **(D)**; *trqa1–1* shown in **(E)**] plants (*n* ≥ 10). To improve seed visibility in **(E)**, siliques were destained with 80% ethanol before imaging. All bar graph data are presented as mean ± SEM. Differences between mutant or transgenic lines and their corresponding WT controls were determined using Student’s *t*-test: *P< 0.05; **P< 0.01.

Unlike *QQS*, which does not measurably affect plant growth, morphology, or yield (Li et al., 2009; Li and Wurtele, 2015; Li et al., 2015; Qi et al., 2019; O’conner et al., 2021; Tanvir et al., 2022a, b), *TRQA1* overexpression slightly reduced plant biomass and seed yield (**Figure 4**; **Supplementary Figure 3**). The growth reduction associated with *TRQA1* overexpression was evident across multiple growth stages and resulted in a significant decrease in biomass relative to Col-0 (19% reduction, *P<* 0.05). In contrast, *trqa1* did not show a detectable difference in growth compared with WT (**Figure 4**; **Supplementary Figure 3**). RT-qPCR confirmed *TRQA1* transcript abundance in the transgenic and mutant lines was altered as expected, with increased transcript levels in *TRQA1-OE* and reduced levels in the *RNAi* and *trqa1* lines (**Figure 1C**).

Consistent with the growth phenotype, *TRQA1-OE* plants also produced fewer seeds per silique and had a significantly lower total seed yield than Col-0 (8% fewer seeds per silique, *P<* 0.05; 14% lower seed yield, *P<* 0.01; **Figures 4D, E**; **Supplementary Figure 3**). In *trqa1* T-DNA insertion lines, the number of seeds per silique was unchanged, and total seed yield was slightly reduced, although this difference was not statistically significant (**Figures 4D, E**; **Supplementary Figure 3**).

Previous work showed that *TRQA1* overexpression increases chlorophyll under iron-deficient conditions (Grillet et al., 2018). Under normal growth conditions, we likewise observed increased total chlorophyll content in *TRQA1-OE* plants (**Figure 4C**). This increase was attributable specifically to higher chlorophyll b levels, whereas chlorophyll a content remained unchanged despite altered *TRQA1* expression (**Figure 4C**). By contrast, *trqa1* plants did not differ significantly from the WT in total chlorophyll content.

Together, these data show that *TRQA1* overexpression affects whole-plant performance in addition to metabolic composition, reducing growth and yield while increasing chlorophyll accumulation. To better understand the biological context in which *TRQA1* functions, we next examined its spatial and temporal expression pattern.

### *TRQA1* is broadly expressed throughout Arabidopsis development

**Figure 5:**
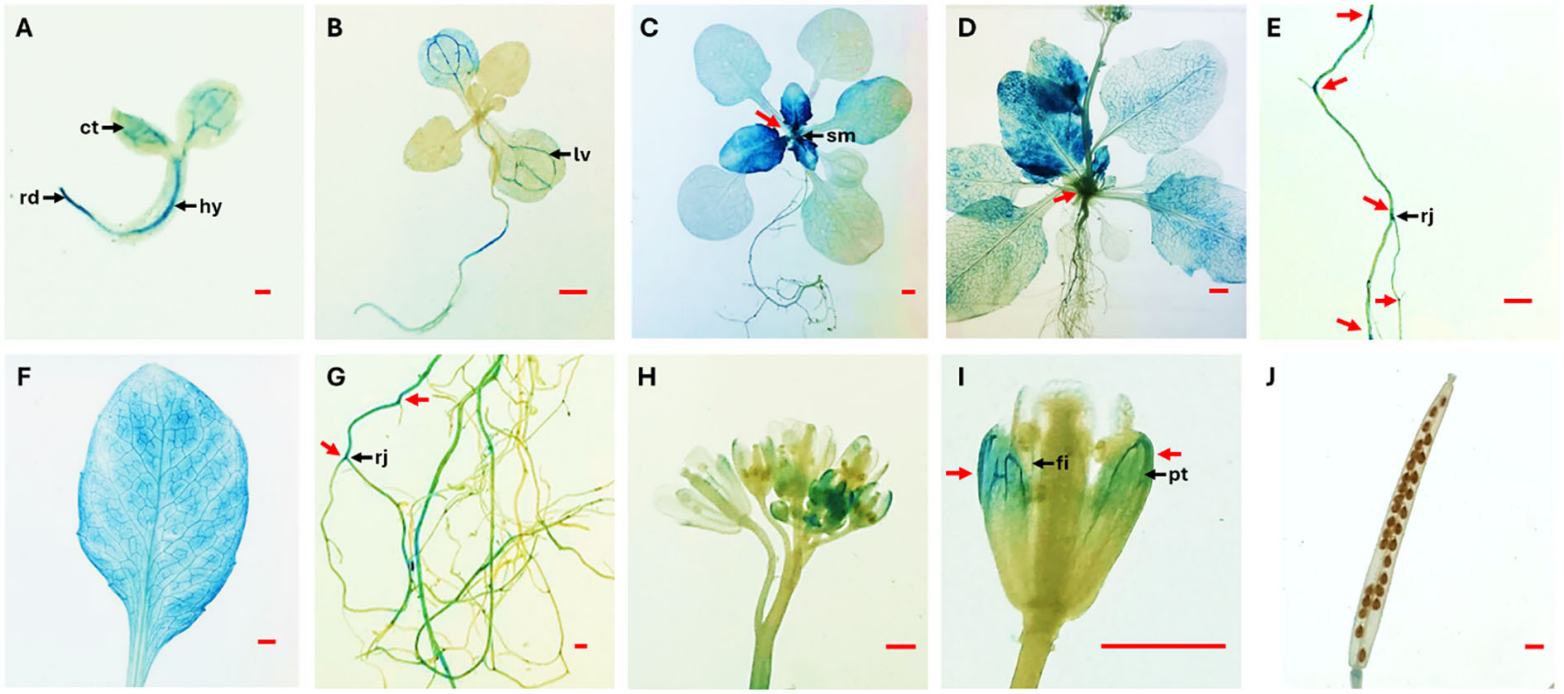
*TRQA1* expression across Arabidopsis organs and developmental stages. GUS activity was analyzed in transgenic plants carrying a *TRQA1* promoter–GUS/GFP fusion construct. Five independent lines were examined, with at least three plants per line evaluated at each developmental stage. Representative images are shown for **(A)** 2 days after imbibition, **(B)** 1 week after imbibition, **(C)** 2 weeks after imbibition, **(D, E)** 3 weeks after imbibition, and **(F–J)** 4 weeks after imbibition. The leaf shown in **(F)** is the ninth rosette leaf. Regions of strong expression are indicated by red arrows. ct, cotyledon; fi, filament; hy, hypocotyl; lv, leaf vein; pt, petal; rd, radicle; rj, root junction; sm, shoot meristem. Scale bars = 1 mm.

*TRQA1* promoter activity was detectable as early as 2 days after imbibition in cotyledons, hypocotyls, and radicles (**Figure 5A**). The strongest expression was observed in the radicle, particularly in the root meristem region, with additional staining in cotyledons, especially along the veins, and in hypocotyls, where darker staining was associated with vascular tissues. This pattern persisted through 1 week after imbibition, when cotyledons continued to express *TRQA1*, whereas recently emerged young leaves showed little or no detectable expression (**Figure 5B**).

By 2 weeks after imbibition, GUS staining in leaves shifted markedly. Younger rosette leaves and the shoot meristem showed stronger staining than mature leaves and cotyledons, and this pattern persisted until senescence (**Figure 5**). At 3 weeks after imbibition, GUS staining in the leaves was especially pronounced in leaf veins (**Figure 5**).

Throughout development, GUS activity was detected throughout the root system. Particularly strong staining was observed at root branching points 3 to 4 weeks after imbibition (**Figure 5**). In flower buds, *TRQA1* expression was initially low, but became detectable in petals and filaments after flower opening (**Figure 5**). In contrast, little or no GUS activity was observed in other floral organs, including the pedicel, sepals, anther, stigma, and styles. During silique development, *TRQA1* expression increased slightly in the pedicel, whereas no detectable GUS activity was observed in developing siliques (**Figure 5J**).

The strong expression of *TRQA1* in aerial tissues, together with comparable expression levels in roots and shoots, is consistent with previous reports (Grillet et al., 2018; Hirayama et al., 2018). Publicly available expression data also support the broad developmental expression of *TRQA1*, with relatively high transcript abundance in rosette leaves, hypocotyl, vegetative shoot apex, imbibed seeds, roots, and cotyledons (Schmid et al., 2005; Winter et al., 2007); **Supplementary Figures 4**, **S5**). Overall, these results indicate that *TRQA1* is broadly expressed across organs and developmental stages, with stronger expression in vascular tissues, root meristems, young leaves, and root branching sites.

Given this broad expression pattern, we next investigated the subcellular localization of the TRQA1 protein to gain further insight into its possible functions.

### TRQA1 localizes to chloroplasts, the plasma membrane, and the cytosol

To determine the subcellular localization of the TRQA1, we generated a translational fusion in which the *TRQA1* promoter and the coding sequence for the full-length TRQA1 protein were fused to GFP. We reasoned that any native targeting information present in TRQA1 would direct the fusion protein to the same cellular compartments as the endogenous protein. Leaf and root tissues from five independent *TRQA1–GFP* transgenic lines were examined by confocal fluorescence microscopy.

**Figure 6:**
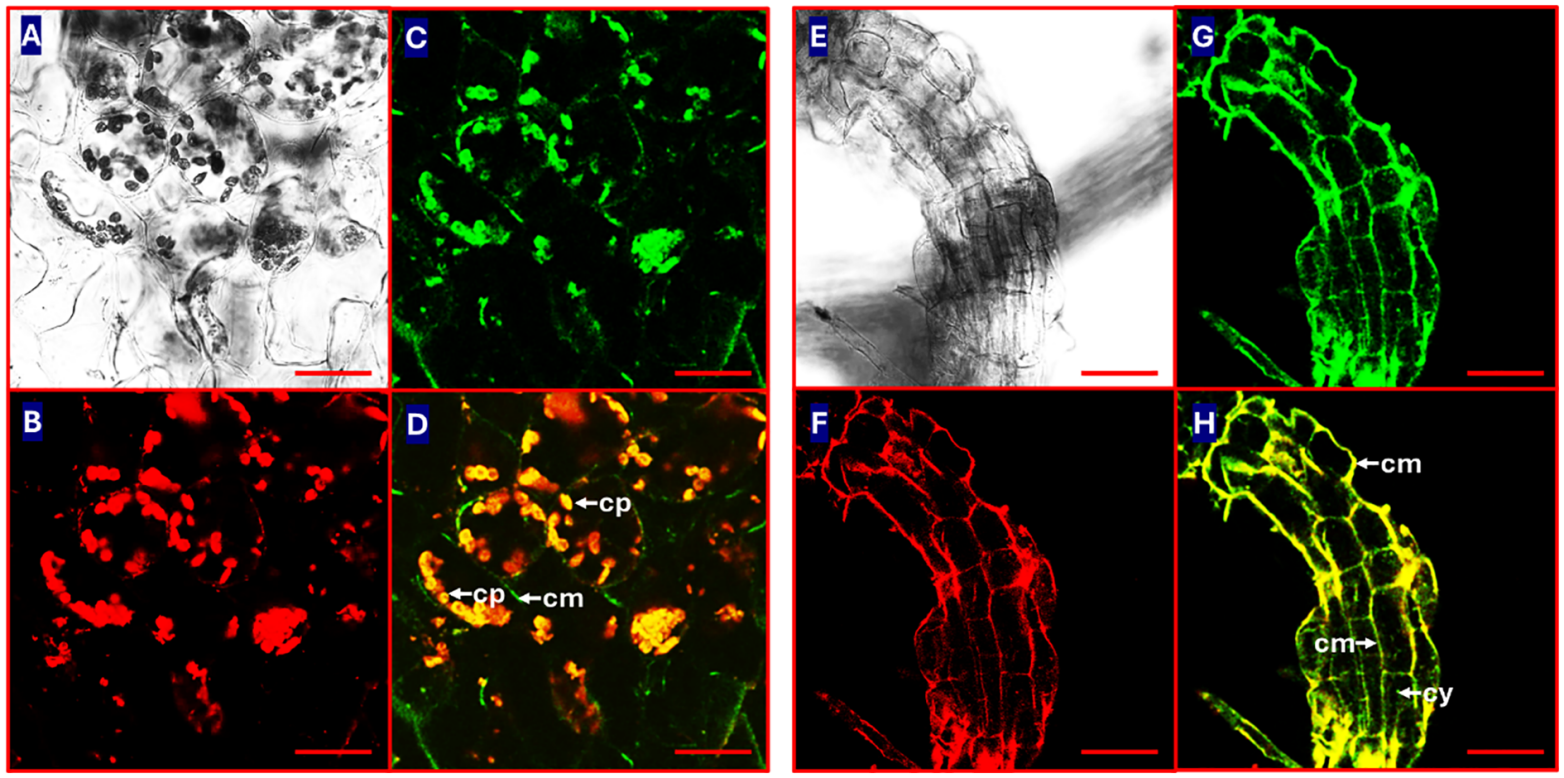
Subcellular localization of TRQA1–GFP in Arabidopsis leaf and root tissues. Fully expanded leaves and roots from transgenic Arabidopsis plants were examined by confocal microscopy 3 weeks after imbibition. Fifteen leaves and fifteen root samples from five independently transgenic lines were analyzed, with three samples of each tissue type evaluated per line. **(A–D)** leaf tissue; **(E–H)** root tissue. **(A, E)** bright-field images; **(B, F)** red autofluorescence; **(C, G)** GFP fluorescence; **(D, H)** merged images. cm, cell membrane; cp, chloroplast; cy, cytosol. Scale bars = 25 µm.

In leaf tissue, the TRQA1–GFP fusion protein was detected in chloroplasts, the plasma membrane, and, to a lesser extent, the cytosol (**Figure 6**). In root tissue, the fusion protein was detected primarily in the cell membrane and cytosol (**Figure 6**). The observed cytosolic localization is consistent with a previous report (Grillet et al., 2018).

These localization patterns are compatible with a role for TRQA1 in metabolic and signaling processes. To further explore the regulatory context of *TRQA1*, we next analyzed its promoter for transcription factor-binding sites and *cis*-regulatory elements.

### Regulatory motif analysis suggests broad control of *TRQA1* by metabolic and stress-related pathways

**Figure 7:**
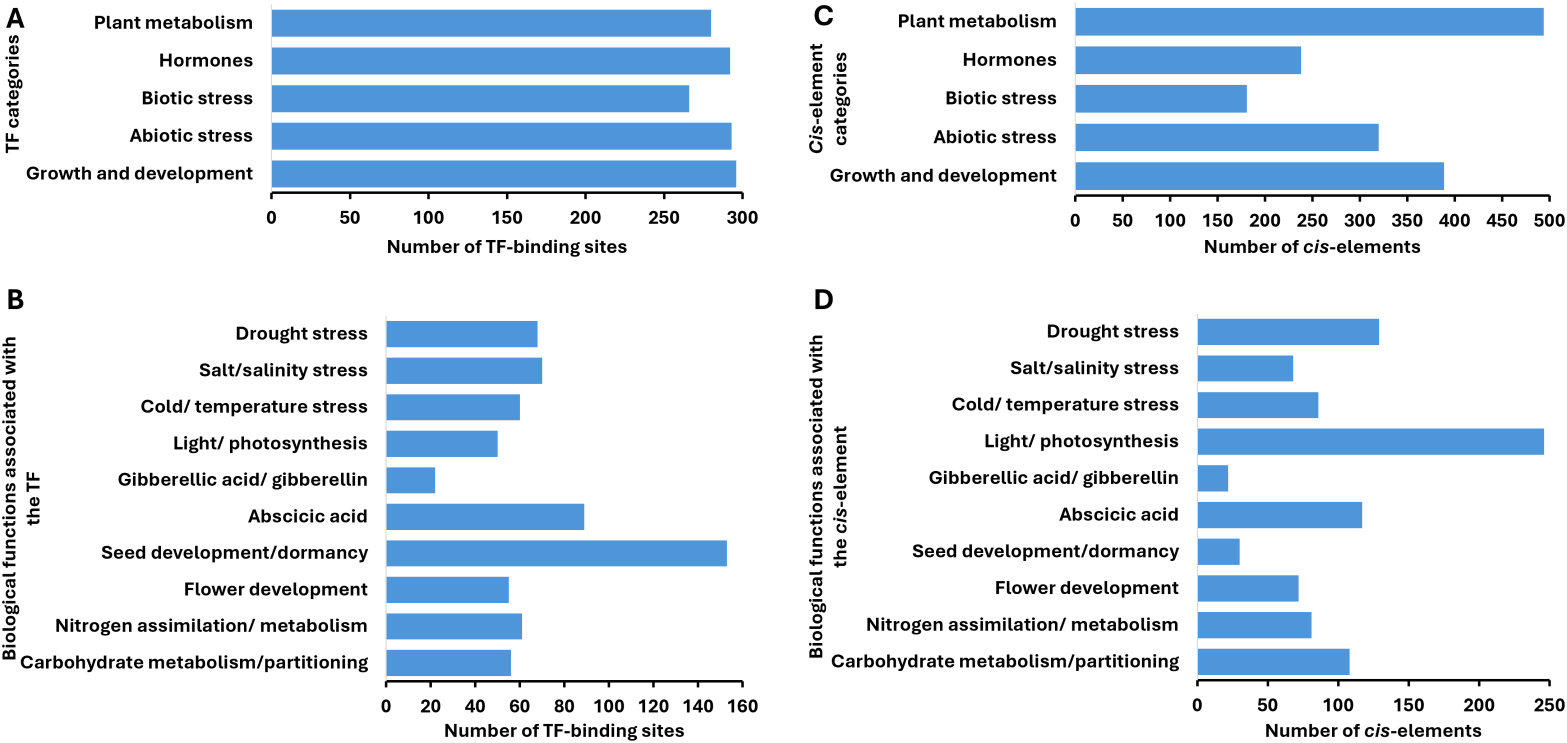
Biological functions associated with predicted TF-binding sites and *cis*-elements in the *TRQA1* promoter. Biological functions were classified into five major categories for all predicted TF-binding sites **(A)** and *cis*-elements **(B)**. The functions associated with the most abundant TF-binding sites **(C)** and *cis*-elements **(D)** are shown separately. Putative TF-binding sites were identified using PlantRegMap (*P* ≤ 1 × 10^-4^), and *cis*-elements were identified using PlantCARE and PLACE, with the 2-kb upstream region of the *TRQA1* as the query sequence.

Analysis of the *TRQA1* promoter region, defined as the 2-kb sequence upstream of the start codon, using PlantRegMap identified 296 putative transcription factor (TF)-binding sites representing 31 TF families (*P* ≤ 1 × 10^-4^; **Supplementary Tables 3**, **S4**). Based on their reported biological functions, these TFs were grouped into five major categories: metabolism, growth and development, abiotic stress, biotic stress, and hormone signaling. Of the 296 predicted sites, 280 were associated with TFs involved in plant metabolism, including carbon and nitrogen uptake and allocation, sugar and carbohydrate transport, storage and metabolism, and protein synthesis and storage (**Supplementary Table 3**; **Figure 7**). Nearly all predicted TF-binding sites (293) were also linked to responses to abiotic stresses such as drought, temperature fluctuations, flooding, and salinity, whereas 266 were associated with biotic stress responses, including defense against pests and pathogens (**Supplementary Table 3**; **Figure 7**).

The most abundant TF families in the *TRQA1* promoter were WRKY (80 sites), bZIP (30 sites), MYB (29 sites), TCP (23 sites), and Dof (18 sites). These TF families are well known for their roles in stress responses and metabolic regulation (**Supplementary Table 3**). These findings suggest that *TRQA1* is subject to complex transcriptional regulation at the intersection of metabolism, development, and environmental response.

To further characterize the regulatory potential of the promoter, we performed *cis*-element analysis using PlantCARE and PLACE. Combined analysis identified 89 distinct *cis*-elements, occurring a total of 586 times within the 2-kb promoter region (**Supplementary Table 5**; **Figure 7**). Of these, 494 were associated with metabolic functions, including motifs linked to RUBISCO, storage proteins, patatin, amylase and isoamylase, ribosomal protein synthesis, protease induction, root nodulation, carbohydrate metabolism and degradation, sugar repression, the Krebs cycle, glycolysis, photosynthesis, and chlorophyll a/b binding proteins (**Supplementary Table 5**; **Figure 7**). In addition, 320 *cis*-elements were associated with abiotic stress responses, and 181 were linked to biotic stress responses.

To complement the promoter analyses, we also examined genes co-expressed with *TRQA1* using Expression Angler (Toufighi et al., 2005). This analysis identified multiple co-expressed genes associated with stress responses, plant defense, and metabolic processes (**Supplementary Table 6**), further supporting a multifaceted role for *TRQA1*. It should be noted that these predictions were generated using default parameters and were not compared against a background set of randomly selected Arabidopsis promoters; the biological significance of individual motifs should therefore be interpreted as hypothesis-generating rather than statistically enriched.

Because the promoter analysis suggested potential integration of metabolic and regulatory pathways, we next explored whether TRQA1 might be connected to the QQS–NF-YC4 module through predicted protein–protein interactions.

### Computational predictions suggest a putative link between TRQA1 and the QQS-NF-YC4 module through MYB103

The secondary structure of the TRQA1 protein was predicted using AlphaFold and I-TASSER, and structural composition was analyzed with PROMOTIF (**Supplementary Figure 6**; **Supplementary Table 7**). Across all prediction platforms, TRQA1 was predicted to consist predominantly of loops (76-82%) and α-helices (18-24%).

We first used AlphaFold2-Multimer (Jumper et al., 2021; Evans et al., 2022) through the ColabFold platform (Mirdita et al., 2022) to test potential direct interactions between TRQA1 and QQS, and between TRQA1 and NF-YC4 (**Supplementary Figure 7**). For both pairs, the model predicted very weak to no interaction, as indicated by AlphaFold confidence scores (pLDDT) below 70 and high Predicted Aligned Error (PAE) values. These results suggest that TRQA1 is unlikely to interact directly with either QQS or NF-YC4. As a positive control, reanalysis of the previously demonstrated QQS–NF-YC4 interaction (Li et al., 2015) yielded a strong predicted interaction, with pLDDT = 100 and PAE = 0 (**Supplementary Figure 7**).

**Figure 8:**
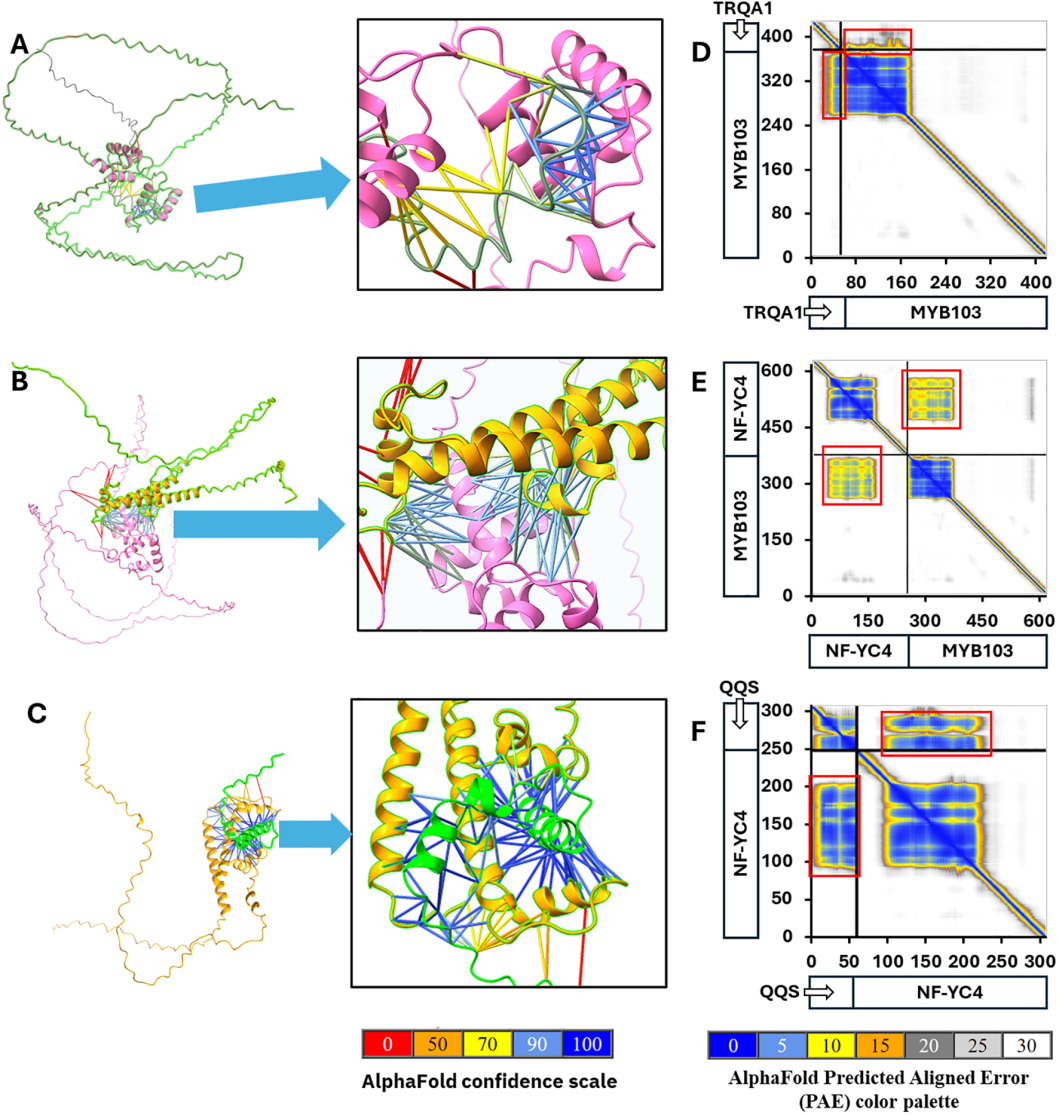
AlphaFold2-Multimer prediction of protein interactions among TRQA1, MYB103, NF-YC4, and QQS. Protein complex structures were predicted using ColabFold with AlphaFold2-Multimer predictions (Jumper et al., 2021; Evans et al., 2022; Mirdita et al., 2022). The best-ranked models for the predicted TRQA1–MYB103 **(A)**, NF-YC4–MYB103 **(B)**, and QQS–NF-YC4 **(C)** complexes are shown. Structural views of residues within 5 Å were generated in ChimeraX (Meng et al., 2023). Insets show the predicted interaction interfaces and AlphaFold confidence for the corresponding secondary structure regions. **(D–F)** Heat maps show the predicted aligned error (PAE) for the TRQA1–MYB103 **(D)**, NF-YC4–MYB103 **(E)**, and QQS–NF-YC4 **(F)** complexes. Red boxes indicate the predicted interaction regions. Axes represent amino acid positions.

We next expanded the analysis to include additional candidate proteins selected on the basis of previous studies and promoter-region analysis of *QQS* (**Supplementary Tables 8**, **S9**). Among the proteins, MYB103 (AT1G63910) emerged as the most likely interactor of TRQA1. AlphaFold2-Multimer predicted the TRQA1–MYB103 interaction with the highest confidence and lowest error among the tested pairs, with pLDDT values close to 100 and PAE values close to 0 specifically within the predicted interaction region (**Figures 8A, D**). These results suggest that TRQA1 and MYB103 may interact directly.

Because MYB103 has previously been shown to positively regulate *QQS* expression (Öhman et al., 2013), we then checked whether MYB103 might provide an indirect connection between TRQA1 and the QQS–NF-YC4 module. Interestingly, regions of MYB103 and NF-YC4 were predicted to interact with high confidence (pLDDT > 90, PAE ≈ 0–5; **Figure 8B**). The model further suggested that the N-terminal region of MYB103 predicted to interact with TRQA1 also interacts with NF-YC4, and that the region of NF-YC4 predicted to interact with MYB103 overlaps with the region that interacts with QQS (**Figure 8**). These predictions support a model in which TRQA1 may connect indirectly to the QQS–NF-YC4 regulatory module through MYB103.

Consistent with this possibility, STRING analysis (Szklarczyk et al., 2020) identified additional candidate associations between TRQA1 and proteins involved in metabolism and nutrient/metal homeostasis (**Supplementary Figure 8**). Together, these computational analyses generate testable hypotheses regarding potential mechanistic connections linking TRQA1 to transcriptional and metabolic regulatory networks in plants.

## Discussion

### *TRQA1* is a Brassicaceae-restricted regulator associated with metabolic traits opposite to those of QQS

In this study, we identified *TRQA1* as a Brassicaceae-restricted gene whose expression pattern is opposite to that of the Arabidopsis orphan gene *QQS*. *TRQA1* expression increased when *QQS* expression was reduced, and the two genes also showed contrasting diurnal expression patterns, with *TRQA1* expression at relatively higher levels during the light period and *QQS* showing higher expression during the dark period. Although these observations do not establish a direct regulatory relationship, they support an inverse association between the two genes and are consistent with their opposite effects on metabolic composition. Specifically, *TRQA1* promoted starch accumulation and reduced protein content, whereas *QQS* has been shown to reduce starch and increase protein accumulation (Li et al., 2009; Li and Wurtele, 2015; Li et al., 2015). Because starch metabolism is tightly linked to photoperiod and carbon status, the opposing diel expression patterns of *TRQA1* and *QQS* may reflect their participation in different phases of carbon allocation.

Our phenotypic analyses further showed that *TRQA1* overexpression reduced plant biomass and seed yield, whereas *TRQA1* knockdown or T-DNA insertion had little or no detectable effect on these agronomic traits. This asymmetric phenotype suggests that elevated *TRQA1* expression perturbs metabolic balance sufficiently to affect whole-plant performance, whereas reduced expression may be partially buffered by compensatory mechanisms. These findings distinguish *TRQA1* from *QQS*, which has been shown to alter composition without measurable penalties on growth or yield in multiple systems (Li et al., 2009; Li and Wurtele, 2015; Li et al., 2015; Qi et al., 2019; O’conner et al., 2021; Tanvir et al., 2022a, b). Together, the contrasting effects of *TRQA1* and *QQS* support the idea that lineage-restricted genes can occupy distinct regulatory positions within metabolic networks, even when they are functionally associated.

### *TRQA1* links metabolic composition with chlorophyll accumulation and plant performance

A notable feature of *TRQA1* overexpression was the increase in total chlorophyll content, driven primarily by chlorophyll b, without a corresponding increase in chlorophyll a. This result is consistent with previous work showing elevated chlorophyll under iron-deficient conditions in *TRQA1*-overexpressing plants (Grillet et al., 2018). Because iron is essential for chloroplast function, chlorophyll biosynthesis, and nitrogen metabolism (Li et al., 2021), the chlorophyll phenotype observed here may reflect broader effects of *TRQA1* on plastid-associated metabolic processes. The preferential increase in chlorophyll b is consistent with a potential expansion of light-harvesting antenna complexes, as chlorophyll b is specifically associated with peripheral light-harvesting complexes and its accumulation necessarily reduces the chlorophyll a/b ratio (Wu et al., 2020; Khan et al., 2023). Under the growth conditions used here, an expanded antenna could lead to excess light absorption relative to photochemical capacity, potentially imposing a photoprotective burden by increasing non-photochemical quenching or reactive oxygen species production rather than enhancing net carbon assimilation. This would provide a plausible mechanistic link between the chlorophyll b increase and the biomass decline observed in *TRQA1-OE* plants, although direct measurements of photosynthetic rate, stomatal conductance, and chlorophyll fluorescence parameters, including Fv/Fm, ΦPSII, and NPQ, will be required to distinguish between enhanced light utilization and photodamage as the underlying cause (Bielczynski et al., 2020).

At the same time, *TRQA1* overexpression reduced biomass and seed yield. This indicates that increased chlorophyll content did not translate into enhanced growth under our conditions. One possible explanation is that altered carbon partitioning toward starch, together with reduced protein accumulation, may limit efficient conversion of photosynthate into biomass and reproductive output. More broadly, the simultaneous effects on chlorophyll, starch, protein, growth, and yield suggest that *TRQA1* participates in coordinating metabolic status with physiological performance, rather than affecting a single downstream trait in isolation.

An important context for interpreting the metabolic phenotypes reported here is the established role of *TRQA1* as *FEP3*/*IMA1*, a phloem-localized peptide that activates iron deficiency-responsive genes and regulates systemic iron distribution in Arabidopsis (Grillet et al., 2018; Hirayama et al., 2018; Cao et al., 2024). Iron serves as an essential cofactor for key enzymes in both photosynthetic electron transport and nitrogen assimilation, including components of the electron transport chain, nitrite reductase, and glutamate synthase, all of which depend on iron-sulfur clusters or heme-iron for activity (Nasar et al., 2022; Yang et al., 2023; Ali et al., 2025; Haque et al., 2026). It is therefore plausible that the metabolic phenotypes observed in *TRQA1-OE* plants are at least partially downstream of altered iron availability or distribution in photosynthetic tissues, rather than exclusively the result of direct transcriptional regulation of carbon–nitrogen partitioning. The selective increase in chlorophyll b is consistent with this possibility, as iron deficiency has been shown to affect chlorophyll synthesis and nitrogen metabolism in a coordinated manner (Li et al., 2021). At the same time, the reciprocal starch and protein phenotypes observed in reduced-*TRQA1* lines under normal iron supply conditions suggest that TRQA1 may also have a more direct role in metabolic regulation. Whether the iron-homeostasis and carbon–nitrogen allocation functions of *TRQA1* are mechanistically linked, operate in parallel, or represent context-dependent manifestations of the same underlying activity remains an important open question.

### Broad expression and subcellular localization are consistent with a multifunctional role for TRQA1

The *TRQA1* promoter–GUS analyses showed that *TRQA1* is broadly expressed across developmental stages and organs, with particularly strong expression in cotyledons, root meristems, vascular tissues, young leaves, and root branching points. This widespread but spatially patterned expression suggests that *TRQA1* may function in multiple developmental contexts, especially in tissues with high metabolic activity, active transport, or developmental plasticity. Strong expression in vascular tissues and root meristems is consistent with a possible role in nutrient allocation and growth-associated metabolism, whereas expression in young leaves and shoot meristems suggests involvement in developing source tissues and vegetative growth.

Subcellular localization of TRQA1–GFP to chloroplasts, the plasma membrane, and the cytosol further supports a multifunctional role. Chloroplast localization is particularly relevant given the starch and chlorophyll phenotypes, because chloroplasts are the major sites of transient starch synthesis and photosynthetic carbon assimilation in leaves. Localization to the plasma membrane and cytosol raises the possibility that *TRQA1* also participates in signaling, transport-associated processes, or protein interactions outside the plastid. Although these data do not establish a specific biochemical function, the combined expression and localization patterns place *TRQA1* in cellular and developmental contexts consistent with broad metabolic regulation.

### TRQA1 shifts carbon and nitrogen allocation toward starch and away from protein accumulation

The clearest functional outcome of altered *TRQA1* expression was its effect on starch and protein accumulation. *TRQA1* overexpression increased starch content and reduced total protein content, whereas *TRQA1* suppression or T-DNA insertion produced the opposite pattern. These reciprocal effects strongly support a role for *TRQA1* in carbon and nitrogen allocation. In addition to the published microarray evidence, our RT-qPCR analysis showed that *QQS* transcript levels changed inversely with *TRQA1* expression in the transgenic and mutant backgrounds used in this study. This reciprocal expression pattern strengthens the conclusion that *TRQA1* and *QQS* are functionally linked, although the directionality and mechanism of this relationship remain to be established. The reciprocal changes in *QQS* transcript levels observed in *TRQA1-OE* and reduced-*TRQA1* lines (**Figure 1D**) suggest that *TRQA1* may influence carbon–nitrogen partitioning at least in part through transcriptional modulation of *QQS*, providing a mechanistic entry point for understanding how altered *TRQA1* expression shifts the balance between starch and protein accumulation.

Importantly, the increase in total protein observed in *TRQA1-RNAi* and *trqa1* lines did not appear to result from preferential accumulation of specific proteins or protein classes, as SDS-PAGE profiles showed no obvious changes in banding patterns. This suggests that *TRQA1* affects overall protein accumulation more broadly, rather than controlling a narrow subset of storage or stress proteins. Such a global shift is consistent with a role in higher-order metabolic allocation rather than terminal biosynthetic steps. Because carbon–nitrogen partitioning is central to plant growth, defense, and stress responses, the metabolic effects of *TRQA1* may have consequences beyond composition alone, including impacts on physiological flexibility and adaptation.

### Promoter architecture suggests integration of metabolic, developmental, and stress-related inputs

The promoter analysis provides additional context for how *TRQA1* may be regulated. The *TRQA1* promoter contains a large number of predicted TF-binding sites and cis-elements associated with metabolism, growth and development, hormone signaling, and biotic and abiotic stress responses. Particularly abundant were motifs linked to WRKY, bZIP, MYB, TCP, and Dof families, all of which have established roles in coordinating developmental and environmental signals with metabolic outputs. The abundance of motifs associated with carbohydrate metabolism, protein storage, photosynthesis, chlorophyll-related functions, and stress responses is consistent with the phenotypes observed in this study.

These promoter features do not prove regulatory function, but they suggest that *TRQA1* could act at a point of convergence between metabolic and stress-responsive pathways. This interpretation is also supported by the co-expression analysis, which identified genes involved in metabolism, defense, and nutrient or metal homeostasis. Together, these findings reinforce the view that *TRQA1* is embedded in a broader regulatory framework that extends beyond primary metabolism alone.

### Interaction modeling suggests an indirect link between TRQA1 and the QQS–NF-YC4 module

Our computational interaction analyses suggest that TRQA1 is unlikely to interact directly with QQS or NF-YC4, but may connect to this regulatory system indirectly through MYB103. AlphaFold2-Multimer predicted a high-confidence interaction between TRQA1 and MYB103, and also supported the interaction between MYB103 and NF-YC4. Because MYB103 has previously been reported to positively regulate *QQS* expression (Öhman et al., 2013), these predictions raise the possibility that MYB103 serves as a bridge between TRQA1 and the QQS–NF-YC4 module.

These structural predictions raise the possibility that MYB103 functions as an intermediate linking TRQA1 to the QQS–NF-YC4 regulatory framework. Rather than acting through direct interaction with QQS or NF-YC4, TRQA1 may influence metabolic output indirectly through MYB103, thereby affecting NF-YC4-associated regulation. Direct experimental validation using Y2H, BiFC, or Co-IP assays will be necessary to determine whether these predicted interfaces operate *in vivo* and whether MYB103 loss of function phenocopies the metabolic effects of *TRQA1* overexpression, as predicted by this model.

Taken together, these observations suggest a plausible, although still speculative, mechanistic basis for the functional opposition between *TRQA1* and *QQS*. *MYB103* has been shown to positively regulate *QQS* expression (Öhman et al., 2013), and our computational predictions suggest that TRQA1 may interact with the N-terminal region of MYB103. If TRQA1 binding alters MYB103 activity or availability, this could reduce MYB103-dependent activation of *QQS* transcription, thereby shift carbon–nitrogen partitioning away from the high-protein, low-starch state promoted by *QQS*. Consistent with this possibility, our RT-qPCR data show that *QQS* transcript levels are reduced in *TRQA1-OE* lines and elevated in *TRQA1-RNAi* and *trqa1* T-DNA insertion lines (**Figure 1D**). Although this model remains to be tested experimentally, it provides a more specific framework for future investigation.

### A proposed working model for TRQA1 function in plant metabolism

**Figure 9:**
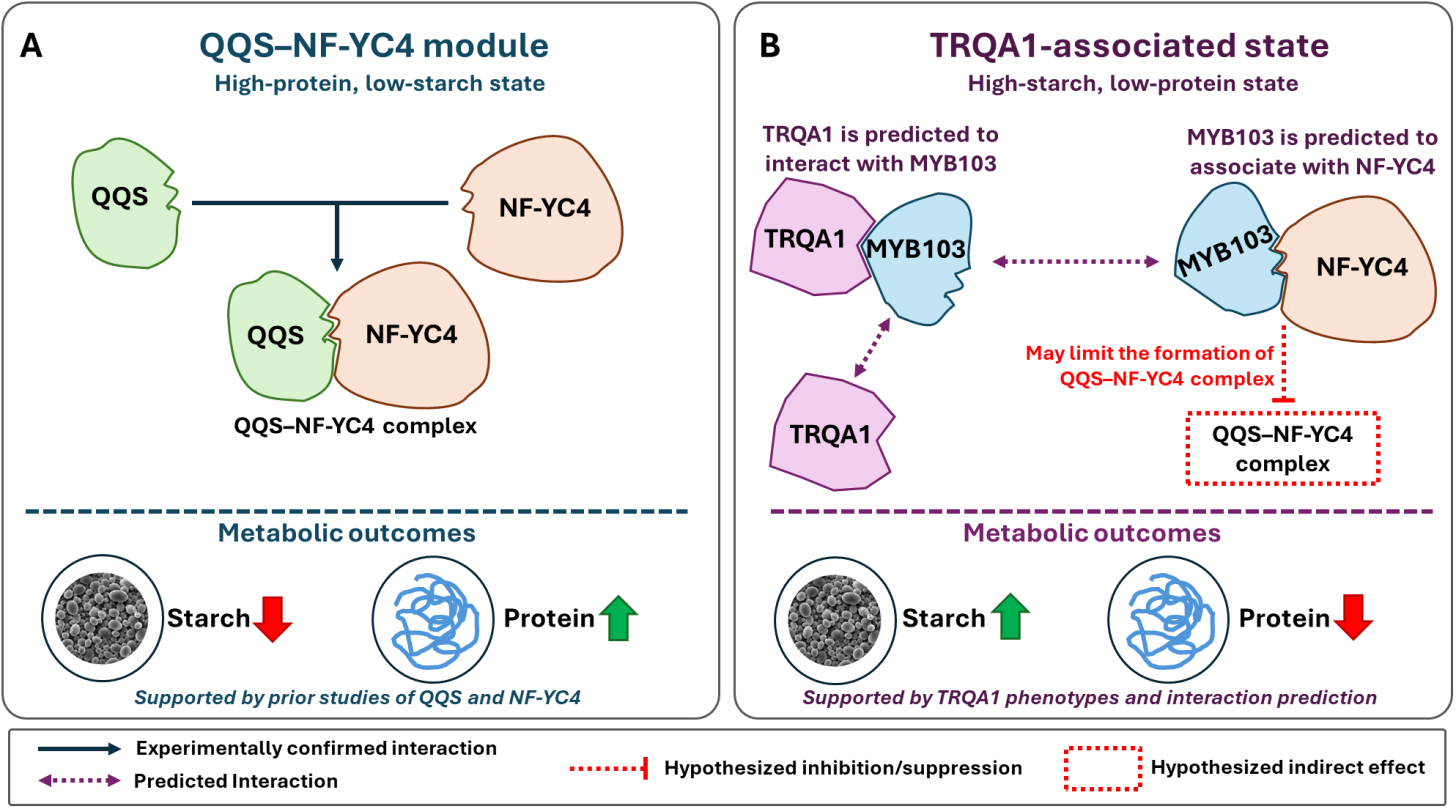
Proposed working model for the predicted role of *TRQA1* in metabolic regulation. The model summarizes experimentally supported phenotypes of TRQA1 and QQS together with predicted protein interactions involving TRQA1, MYB103, and NF-YC4 in the QQS-mediated high-protein, low-starch state **(A)** and the TRQA1-associated high-starch, low-protein state **(B)**.

Based on the genetic, physiological, promoter, localization, and computational interaction data, we propose a working model in which *TRQA1* acts as a negative regulator of the *QQS*-associated high-protein, low-starch state (**Figure 9**). In this model, *TRQA1* promotes starch accumulation and reduces protein accumulation, potentially through interaction with MYB103 and indirect modulation of the QQS–NF-YC4 module. This framework provides a testable, though still speculative, explanation for the opposite expression patterns and metabolic effects of *TRQA1* and *QQS*, while explicitly recognizing that all proposed protein interactions require experimental confirmation.

Several limitations of the present study should be acknowledged. First, phenotypic characterization was performed using T_2_-generation transgenic plants, for which homozygosity was not confirmed beyond PCR-based detection of the transgene and selection-based screening. Although two independent transgenic lines per class exhibited consistent and statistically significant phenotypes, arguing against insertion-specific artifacts, we cannot completely exclude the possibility that hemizygosity or minor positional effects contributed to the observed variation. Second, starch content was measured at a single time point, the end of the light period, when accumulation is maximal. While this approach maximizes sensitivity for detecting genotypic differences, it does not allow formal distinction between increased rates of starch synthesis and reduced rates of nocturnal degradation as the basis for the observed differences in starch accumulation. Third, the proposed regulatory interactions between TRQA1, MYB103, and NF-YC4 are based entirely on computational predictions using AlphaFold2-Multimer and have not been validated by any *in vivo* or *in vitro* protein interaction assay. These predictions should therefore be regarded strictly as hypothesis-generating. In addition, the identification of direct downstream target genes involved in starch synthesis and nitrogen assimilation pathways remains an important unresolved question.

At the same time, several aspects of this model remain unresolved. It is not yet known whether *TRQA1* acts primarily through transcriptional regulation, protein interaction, plastid-associated metabolic processes, or a combination of these mechanisms. Addressing these questions will require direct interaction assays, transcriptomic or proteomic analyses of *TRQA1* perturbation lines, and functional dissection of the predicted regulatory interfaces. Even so, the present study identifies *TRQA1* as a previously uncharacterized regulator of plant metabolic composition whose reduced function increases protein content at the expense of reduced starch accumulation, a trade-off that will need to be addressed before any applied utility can be realized. These findings expand the growing evidence that taxonomically restricted genes can exert important control over conserved physiological processes and define a clear set of experimental priorities for mechanistic and translational follow-up.

## Data Availability

All sequences analyzed in this study are part of the publicly available Arabidopsis thaliana (Col-0) reference genome and can be retrieved from TAIR (https://www.arabidopsis.org) and NCBI under the following locus identifiers: TRQA1 (AT1G47400), QQS (AT3G30720), MYB103 (AT1G63910), and ACT2 (AT3G18780). The TRQA1 promoter analyzed corresponds to the 2-kb region upstream of AT1G47400. The predicted TRQA1 protein structure was obtained from the AlphaFold Protein Structure Database (UniProt accession Q3ECW0). The microarray data reanalyzed here were previously published (Li et al., 2009). No new datasets were generated in this study.
