# Supplementary material for: The Brassicaceae-restricted gene TRQA1, acting opposite to QQS, modulates starch and protein accumulation in Arabidopsis
Source: Front Plant Sci. 2026 Jul 7;17:1862971. doi: 10.3389/fpls.2026.1862971 (PMC13386268; doi:10.3389/fpls.2026.1862971)
Supplement: Supplementary file 1 [file Supplementaryfile1.docx]

Supplementary Material for

TRQA1, a novel regulator of plant metabolism, controls protein and starch content in Arabidopsis

Rezwan Tanvir^1^, Caroline Kercheval^1^, Kelsi White^1^, Emma Koeppen^1^, Mary Virginia Miller^1^, Sharnali Das^1^, Lei Wang^1,2^, Ling Li^1*^

^1^ Department of Biological Sciences, Mississippi State University, Mississippi State, MS, United States, ^2^ College of Life Sciences, Shihezi University, Shihezi, China

*** Correspondence:**Ling Li
liling@biology.msstate.edu

**
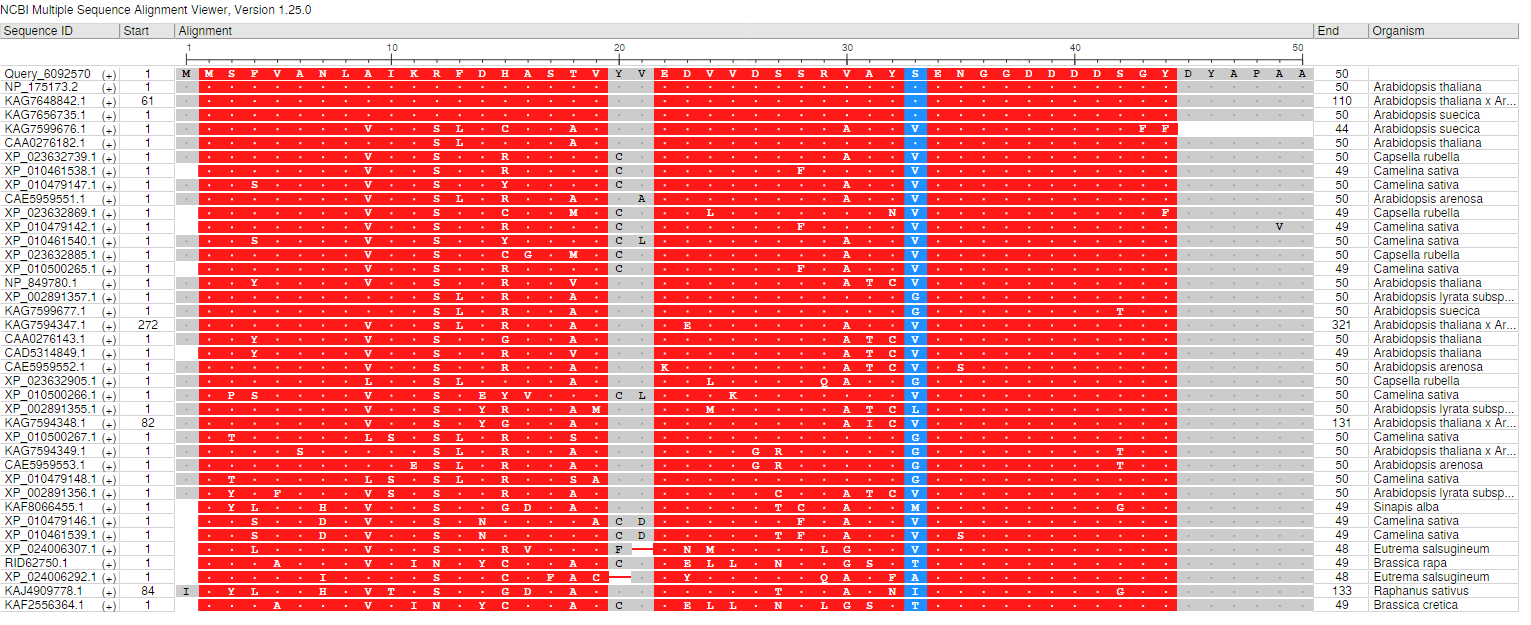
**

**Figure S1**

Protein sequence alignment of TRQA1 with homologous proteins from Brassicaceae species, generated using the NCBI BLAST+ suite (Camacho et al., 2009). Conserved residues are shown in red. Dots indicate identity, and letters indicate amino acid substitutions at the corresponding positions.


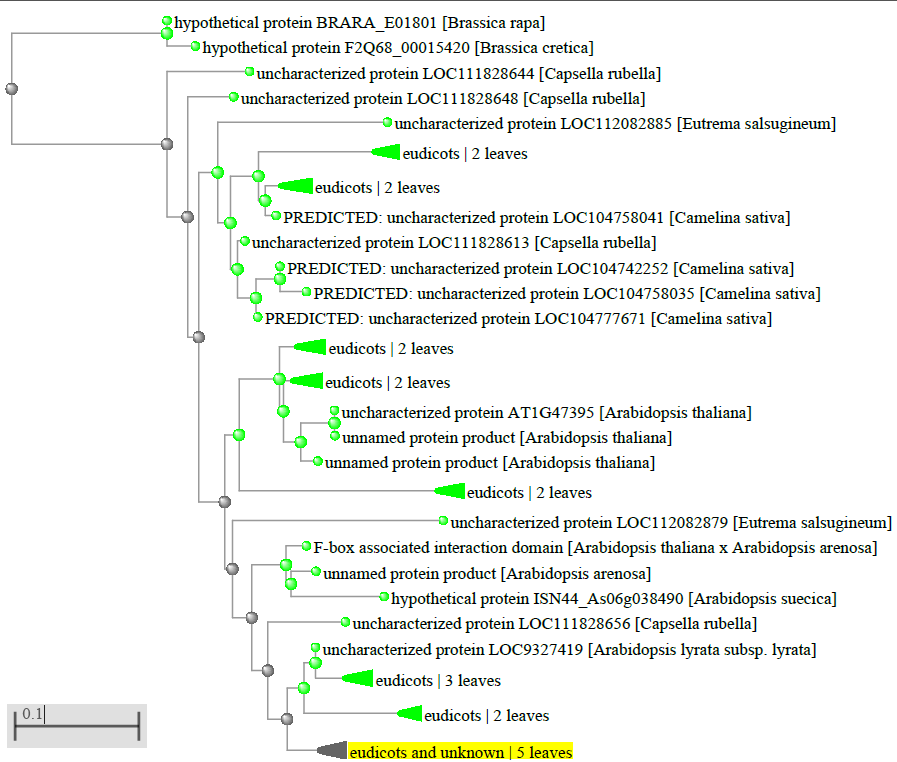


**Figure S2**

Maximum-likelihood phylogenetic tree of TRQA1 and its top-matching proteins from Brassicaceae species, generated using NCBI BLASTp and Tree Viewer (Camacho et al., 2009). The TRQA1 peptide sequence was used as the query, and the top BLASTp matches were included in the analysis. Branch lengths indicate the number of substitutions per site.

**
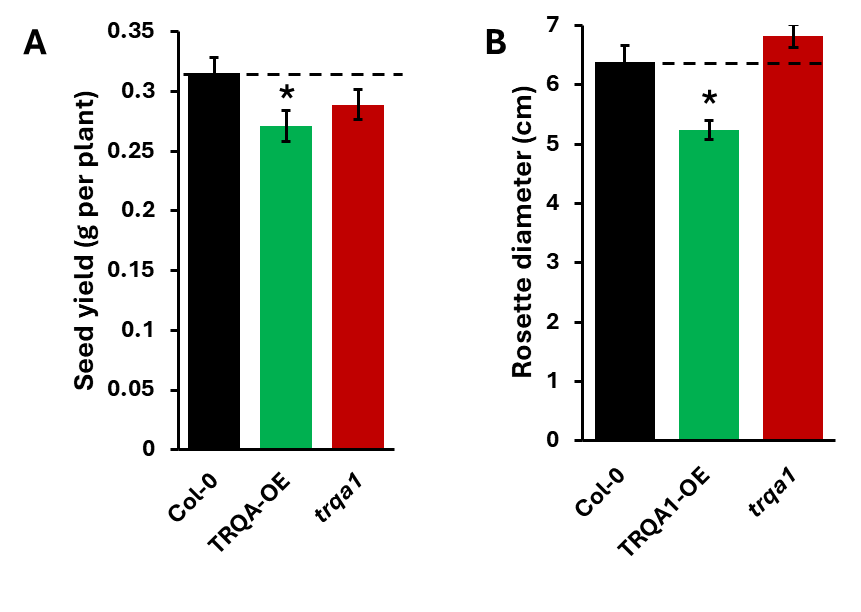
**

**Figure S3**

Effects of *TRQA1* expression on seed yield and rosette diameter. (**A**) Seed yield in grams per mature Col-0, *TRQA1-OE*, and *trqa1* plant (*n* ≥ 6). (**B**) Rosette diameter in centimeters of 3-week-old Col-0, *TRQA1-OE*, and *trqa1* plants (*n* ≥ 6). All bar graph data are presented as mean ± SEM. Differences between transgenic or mutant lines and their corresponding wild-type controls were assessed using Student’s *t*-test, **P* < 0.05.


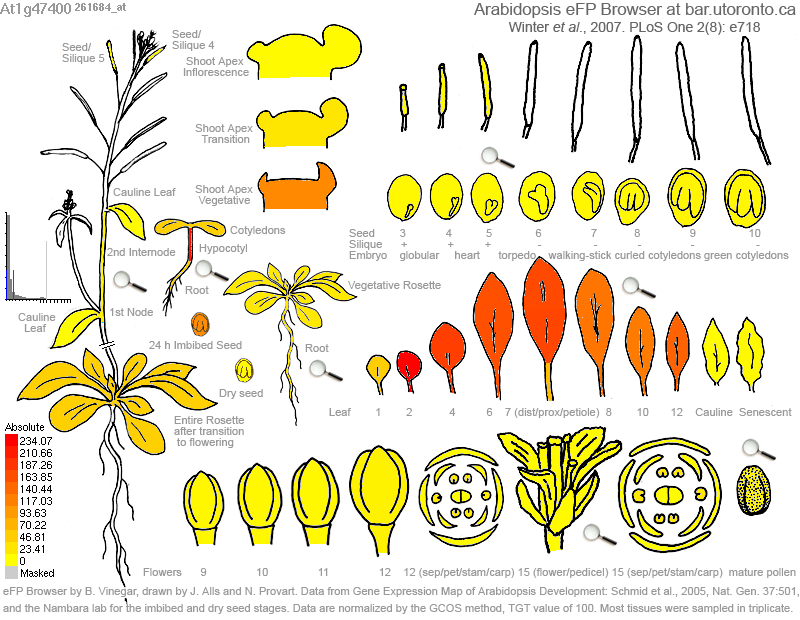


**Figure S4**

Expression of *TRQA1* (At1g47400) across different organs of *Arabidopsis thaliana*, based on publicly available electronic Fluorescent Pictograph (eFP) data (http://bar.utoronto.ca/efp/cgi-bin/efpWeb.cgi?primaryGene=AT1G47400&dataSource=Developmental_Map&modeInput=Absolute) (Schmid et al., 2005; Winter et al., 2007).


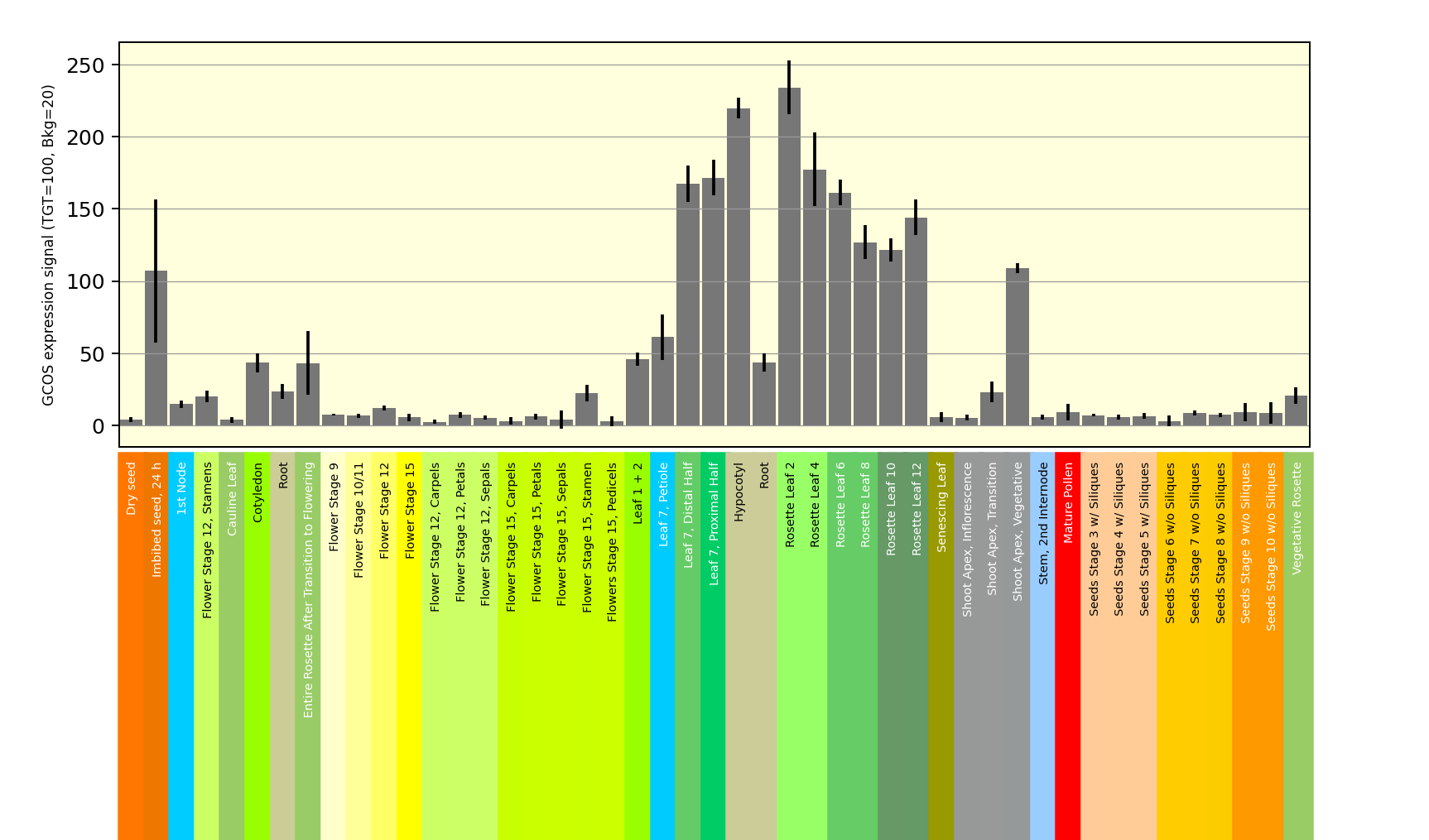


**Figure S5**

Expression signal (GCOS) of *TRQA1* (At1g47400) across different organs of *Arabidopsis thaliana*, based on publicly available microarray data) (<http://bar.utoronto.ca/efp/cgi-bin/efpWeb.cgi?primaryGene=AT1G47400&dataSource=Developmental_Map&modeInput=Absolute>) (Schmid et al., 2005; Winter et al., 2007).

**
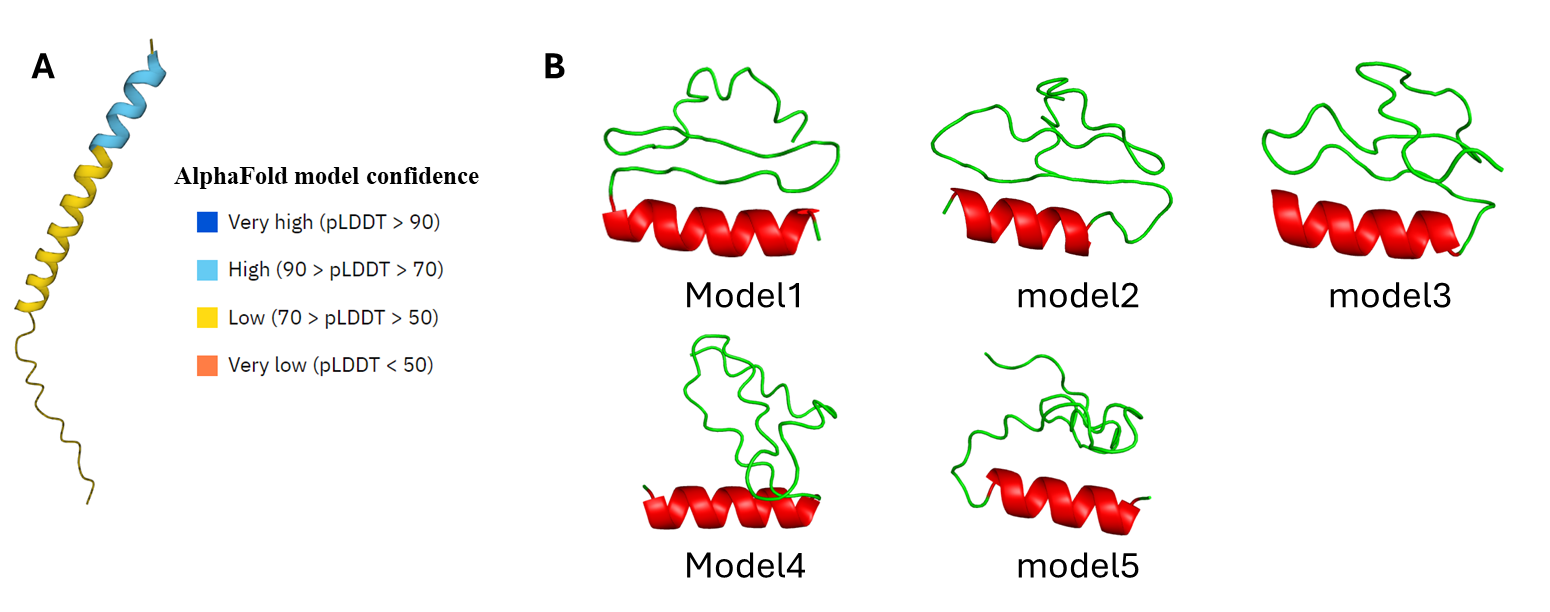
**

**Figure S6**

Predicted secondary structure of TRQA1. Structural models were generated using AlphaFold (**A**) **(**https://alphafold.ebi.ac.uk/entry/Q3ECW0, accessed 8 April 2024) (Jumper et al., 2021; Varadi et al., 2021) and I-TASSER (**B**) (Roy et al., 2010). I-TASSER generated five models, in which α-helices are shown in red and loops in green. Structural images were prepared using PyMol (The PyMOL Molecular Graphics System, Version 3.0; Schrödinger, LLC). The secondary-structure composition of each model is provided in Supplementary Table S7.

**
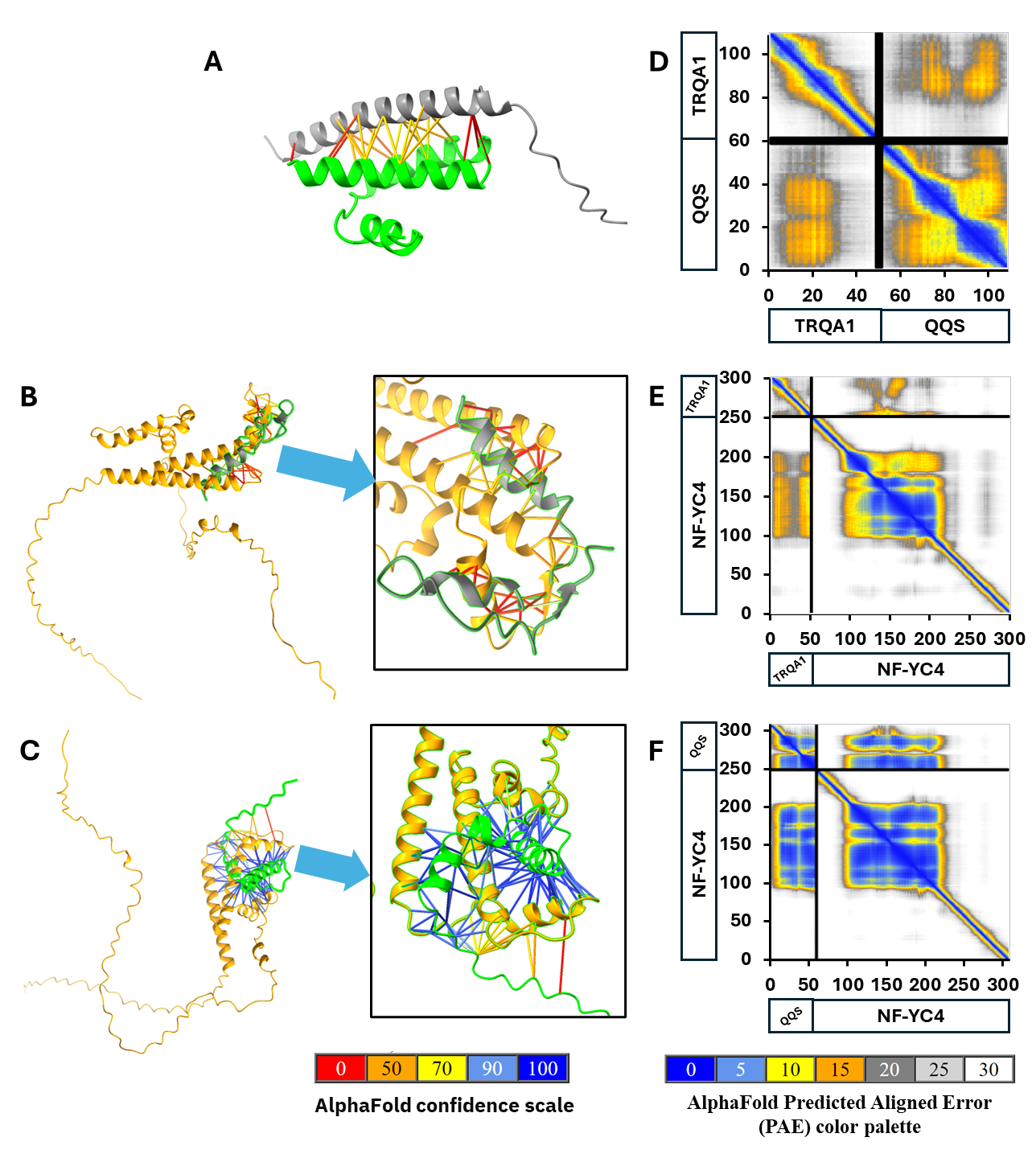
**

**Figure S7**

AlphaFold2-Multimer prediction of protein-protein interactions between TRQA1 and QQS, TRQA1 and NF-YC4, and QQS and NF-YC4. Predictions were generated using ColabFold (Mirdita et al., 2022). The best-ranked models for the TRQA1–QQS (**A**), TRQA1–NF-YC4 (**B**), and QQS–NF-YC4 (**C**) complexes are shown. Structural views of residues within 5Å were generated in ChimeraX (Meng et al., 2023). The TRQA1–NF-YC4 and QQS–NF-YC4 models are enlarged to highlight AlphaFold confidence at the predicted interaction interfaces. (**D–F**) Heat maps show the predicted aligned error (PAE) for the TRQA1–QQS (**D**), TRQA1–NF-YC4 (**E**), and QQS–NF-YC4 (**F**) complexes. Axes represent amino acid positions.

**
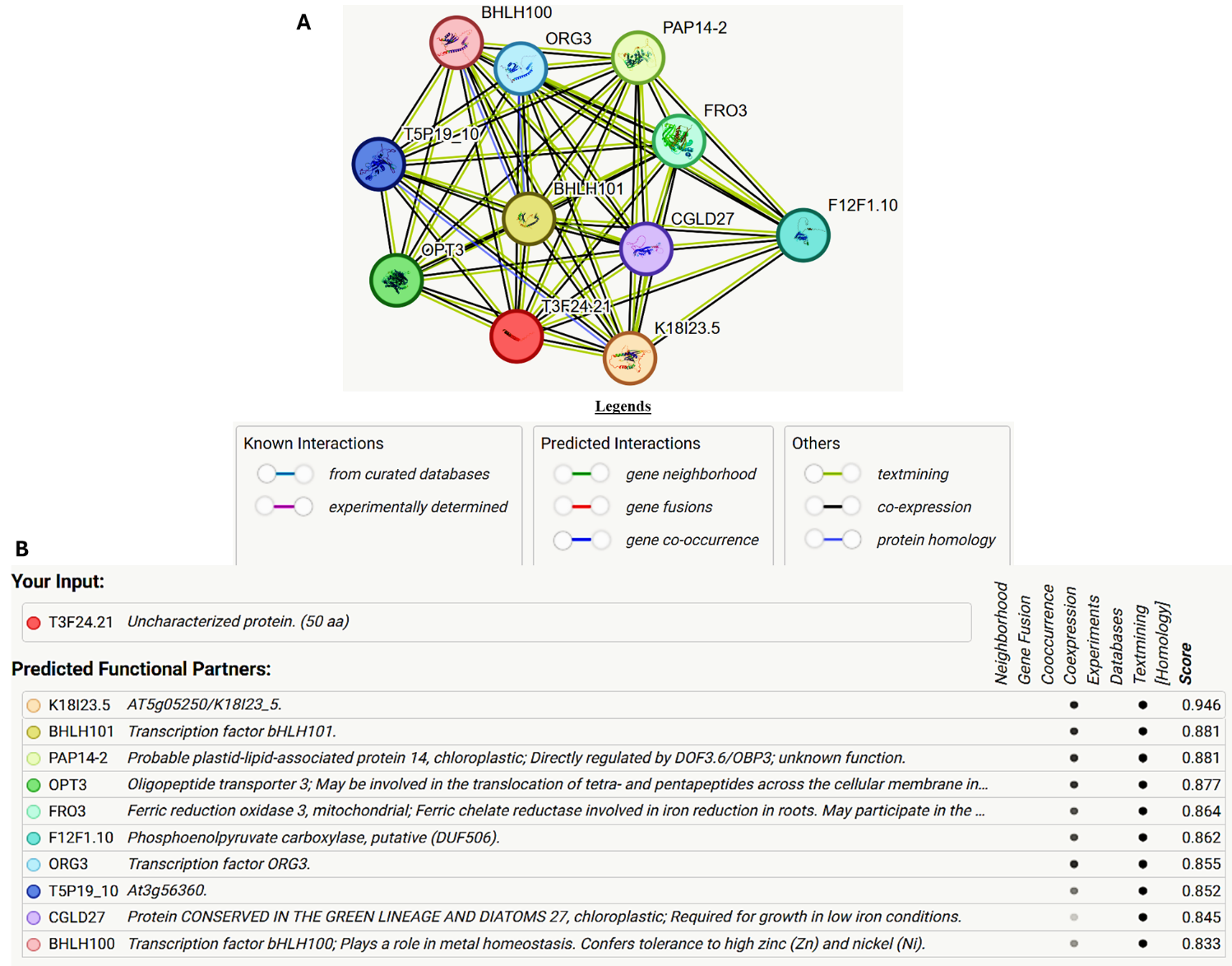
**

**Figure S8**

Functional interaction network of TRQA1 predicted using the STRING Database (Szklarczyk et al., 2019).

**TABLE S1** **Coding and peptide sequences of *TRQA1* (At1g47400) and *QQS* (At3g30720), and the peptide sequence of *AtNF-YC4* (At5g63470).** Start and stop codons in the coding sequences are underlined. All sequences were obtained from TAIR (https://www.arabidopsis.org/) (accessed 20 February 2024).

| Gene | Sequence information |
| --- | --- |
| *TRQA1*  coding  sequence | ATGATGTCTTTTGTCGCAAACTTGGCCATCAAGAGATTTGACCATGCTTCCACCGTGTATGTTGAAGATGTGGTAGATAGTTCTCGAGTGGCATATAGTGAGAATGGTGGTGATGACGATGACAGTGGCTATGATTATGCTCCTGCTGCGTGA |
| TRQA1  peptide  sequence | MMSFVANLAIKRFDHASTVYVEDVVDSSRVAYSENGGDDDDSGYDYAPAA |
| *QQS*  coding  sequence | ATGAAGACCAATAGAGAGCAGGAAATTTACGTTGAAAGAAGCTTCAAACCAAACAATTCAACAATTCAGAATTTGATGGACATTGAAAGGTTCATTTTGCCTCACACTTCTACATCAGGTGTCGCAAGGCTCAAAATGAG GGTCATATCATGGGTCGGGC TTCAGTTCTA CAACTACTGA |
| QQS  peptide  sequence | MKTNREQEIYVERSFKPNNSTIQNLMDIERFILPHTSTSGVARLKMRVISWVGLQFYNY |
| AtNF-YC4  peptide  sequence | MDNNNNNNNQQPPPTSVYPPGSAVTTVIPPPPSGSASIVTGGGATYHHLLQQQQQQLQMFWTYQRQEIEQVNDFKNHQLPLARIKKIMKADEDVRMISAEAPILFAKACELFILELTIRSWLHAEENKRRTLQKNDIAAAITRTDIFDFLVDIVPREEIKEEEDAASALGGGGMVAPAASGVPYYYPPMGQPAVPGGMMIGRPAMDPSGVYAQPPSQAWQSVWQNSAGGGDDVSYGSGGSSGHGNLDSQG |

**TABLE S2** **(File: Supplementary Tables.xlsx) NCBI BLAST+ protein search results for TRQA1 (Camacho et al., 2009).** Using the TRQA1 peptide sequence as the query, 38 homologous proteins were identified, all from species within the Brassicaceae family.

**TABLE S3** **Predicted transcription factor (TF)-binding sites in the 2-kb promoter region upstream of the *TRQA1* start codon.** TF families were grouped according to reported biological functions: M, metabolism; G, growth and development; A, abiotic stress; B, biotic stress; and H, hormone signaling. Binding sites were identified using PlantRegMap (http://plantregmap.gao-lab.org/binding_site_prediction.php) (Tian et al., 2019) with the 2-kb sequence upstream of the *TRQA1* start codon as the query sequence (*P* ≤ 1 × 10^-4^). Additional details are provided in Supplementary Table S4.

| **Number of binding sites** | **TF family** | **TF category** | **Known function** |
| --- | --- | --- | --- |
| 80 | WRKY | M, G, A, B, H | Plays varied roles in plant disease resistance, responses to abiotic stress, coping with nutrient deprivation, senescence, embryogenesis, and various other developmental and hormone-regulated processes (Bakshi and Oelmüller, 2014). |
| 30 | bZIP | M, G, A, B, H | Involved in flower development, seed maturation, dormancy, and senescence, abiotic stresses such as salt damage, drought, cold damage, osmotic stress, mechanical damage, and ABA signal response offering metabolic balance as well as plant response to biological stresses such as insect pests and pathogen infection (Liu et al., 2023). |
| 29 | MYB | M, G, A, B, H | Associated with diverse biological processes, it participates in ABA response and interacts with other transcription factors to regulate biotic and abiotic stresses and influence development, differentiation, metabolism, and defense mechanisms (Ambawat et al., 2013). |
| 23 | TCP | M, G, A, B, H | Regulate various biological processes, including plant development and growth, responses to biotic and abiotic stresses, hormonal pathways, and nitrogen metabolism (Guan et al., 2014; Li et al., 2018a). |
| 18 | Dof | M, G, A, B, H | Regulates hormone signals and diverse biotic or abiotic stress responses and plays a role in numerous plant biological processes, including dormancy, tissue differentiation, carbon and nitrogen assimilation, as well as carbohydrate metabolism (Zou and Sun, 2023). |
| 16 | MYB_related | M, G, A, B, H | Associated with diverse biological processes, they participate in ABA response and interact with other transcription factors to regulate biotic and abiotic stresses and influence development, differentiation, metabolism, and defense mechanisms (Ambawat et al., 2013). |
| 12 | bHLH | M, G, A, B, H | Involved in plant developmental and metabolic processes, such as photomorphogenesis, flowering induction, and secondary metabolite biosynthesis (Wang et al., 2015; Guo et al., 2021b). |
| 11 | G2-like | M, G, A, B, H | Involved in plant growth, development, senescence, biotic and abiotic stresses, chloroplast formation, carbohydrate metabolism, and anthocyanin biosynthesis by hormone signaling pathways (Alam et al., 2022). |
| 9 | HSF | M, G, A, H | Associated with the accumulation of carotenoids, chlorophyll, and anthocyanins, as well as influencing glutathione metabolism, ABA signaling, and secondary metabolites, these factors also play a regulatory role in growth and stress resistance (Jiang et al., 2023). |
| 8 | GATA | M, G, A, B, H | Play a vital role in plant development and growth, including chloroplast development and carbon and nitrogen metabolism regulation. They also suppress gibberellin signaling and respond to both abiotic and biotic stresses (Guo et al., 2021a). |
| 7 | BES1 | M, G, A, B, H | Associated with sugar partitioning, protein metabolism, resistance to bacterial pathogens, hormone signaling, growth and development, and stress response (Li et al., 2018b). |
| 7 | C2H2 | M, G, A, B, H | Regulates metabolism and affects seed storage proteins (total protein) and is associated with growth development processes and resisting biotic and abiotic stress in plants' phytohormone signaling (Feurtado et al., 2011; Liu et al., 2022b). |
| 7 | MIKC_MADS | M, G, A, H | Associated with plant reproduction, development, flower morphogenesis, and responses to different abiotic stresses through hormones (Mou et al., 2022). |
| 4 | C3H | M, G, A, B, H | Plays a crucial role in sugar response, sustaining a balanced ABA/GA response, and being indispensable in regulating plant development, essential for abiotic stress tolerance, including drought and salt, and biotic stress resistance against fungal diseases (Deng et al., 2023). |
| 4 | ERF | M, G, A, B, H | ERFs regulate various crucial processes, including development, hormone regulation, flavonoid synthesis, and responses to both abiotic and biotic stressors by regulating the synthesis of antioxidative metabolites (Wu et al., 2022). |
| 4 | RAV | G, A, B, H | RAV transcription factors are recognized for their involvement in the development and regulation of plant responses to both biotic and abiotic stresses, encompassing interactions with plant pathogens and hormonal pathways (Chen et al., 2021). |
| 3 | B3 | G, A | Involved in embryo maturation, seed development, diverse roles in plant growth and development process, and abiotic stress response (Suzuki and McCarty, 2008; Verma and Bhatia, 2019). |
| 3 | HD-ZIP | M, G, A, H | They demonstrate control over plant growth, development, secondary metabolism, and responses to abiotic stress by modulating hormone regulatory pathways (Li et al., 2022). |
| 3 | Trihelix | M, G, A, B, H | Their function encompasses localized suppression of growth and response to abiotic and pathogenic stresses. They are supposedly involved in metabolism and stress-related hormone pathways (Yang et al., 2023). |
| 2 | BBR-BPC | G, A, H | Involved in plant development, hormone signaling, abiotic stresses, and numerous other biological processes, including circadian oscillation and sex determination (Sahu et al., 2023). |
| 2 | E2F/DP | G, B, H | Stimulate cell proliferation while inhibiting the accumulation of salicylic acid (SA), a well-known regulator of plant immunity (Chandran et al., 2014). |
| 2 | GRAS | G, A, B, H | GATA TFs play diverse roles in functions related to gibberellin, Jasmonate, and light signaling. They contribute to nodule morphogenesis, signal transduction, and the development of roots and shoots while also responding to both biotic and abiotic stresses (Sidhu et al., 2020). |
| 2 | LBD | M, G, A, H | Plays a crucial role in overseeing the development of lateral organs, morphogenesis, hormone signaling, and managing plant metabolism, as well as responding to abiotic stresses such as drought, salt, and cold (Wang et al., 2021). |
| 2 | NAC | M, G, A, B, H | Play a crucial role in various biological processes, such as the development of root and shoot apical meristems, organogenesis, hormone signaling, fruit ripening, and response to biotic and abiotic stresses, as well as influencing fiber and secondary cell wall development, ultimately impacting plant metabolism (Nakashima et al., 2012; Diao et al., 2020). |
| 2 | Nin-like | M, G, A, B, H | Plays a central role in diverse biological processes, encompassing transport, metabolism, hormone signaling, transcriptional activities, and developmental programs in both roots and shoots and are also implicated in responding to both biotic and abiotic stressors (Karve et al., 2016; Liu et al., 2022a). |
| 1 | AP2 | M, G, A, H | Involved in regulating plant growth and development, fruit ripening, defense response, and metabolism and are also implicated in plant abiotic stress and hormone response (Gu et al., 2017). |
| 1 | ARF | G, H | Modulates flowering, embryonic development, seed dormancy, and germination by regulating the gene expression network of auxin signaling (Li et al., 2016). |
| 1 | CAMTA | M, G, A, B, H | Associated with abiotic stress (temperature, drought, UV, salt, and injury, as well as hormones), biotic (SA-mediated immune response), development (a trigger for senescence and death), and regulation of carbohydrate metabolism-related genes (Doherty et al., 2009; Xiao et al., 2021). |
| 1 | CPP | M, G, A | Involved in plant development, growth, and stress responses and regulates the expression of *leghemoglobin c3* gene associated with nitrogen fixation and root nodules (Cvitanich et al., 2000; Nan et al., 2021). |
| 1 | SBP | G, A, H | Has an influence on plant microsporogenesis, flowering, yield, and resistance to stressors (Nan et al., 2021). |
| 1 | SRS | G, A, B, H | Crucial for growth, plant development, phytohormone biosynthesis, and responses to both biotic and abiotic stress (Ma et al., 2022). |

**TABLE S4** **(File: Supplementary Tables.xlsx) Complete list of predicted transcription factor (TF)-binding sites in the 2-kb promoter region upstream of the *TRQA1* start codon and their associated functions.** TF families were classified into five groups based on their reported biological functions. Binding sites were identified using PlantRegMap (http://plantregmap.gao-lab.org/binding_site_prediction.php) (Tian et al., 2019) with the 2-kb region upstream of the *TRQA1* start codon as the query sequence (*P* ≤ 1 × 10^-4^).

**TABLE S5** **(File: Supplementary Tables.xlsx) Complete list of predicted *cis*-elements in the 2-kb promoter region upstream of the *TRQA1* start codon and their associated functions.** *Cis*-elements were classified into five groups based on their reported biological functions. *Cis*-elements were identified using PlantCARE (http://bioinformatics.psb.ugent.be/webtools/plantcare/html/) (Lescot et al., 2002) and PLACE (https://www.dna.affrc.go.jp/PLACE/?action=newplace) (Higo et al., 1999), with the 2-kb region upstream of the *TRQA1* start codon used as the query sequence.

**TABLE S6** **Genes co-expressed with *TRQA1*, identified using Expression Angler (http://bar.utoronto.ca/ntools/cgi-bin/ntools_expression_angler.cgi) (Toufighi et al., 2005), and their functional annotation from TAIR (https://www.arabidopsis.org/index.jsp) (Reiser et al., 2024).** The Pearson correlation coefficient (r-value) for the top 25 hits is provided.

| **Co-expressed genes** | | **r-value** | **Functional annotation** |
| --- | --- | --- | --- |
| At5g53450 | 0.556 | | Contains a lipid-binding FBN domain and a kinase domain. It is induced by osmotic stress and loss of function mutants show decreased germination when grown under osmotic stress conditions. |
| At5g05250 | 0.529 | | Hypothetical protein |
| At5g25190 | 0.524 | | ETHYLENE AND SALT INDUCIBLE 3 encodes a member of the ERF (ethylene response factor) subfamily B-6 of ERF/AP2 transcription factor family. |
| At2g02950 | 0.499 | | Encodes a basic soluble protein which can independently bind to either PHYA or PHYB, regardless of whether the phytochromes are in the Pr or Pfr state. PKS1 can be phosphorylated by oat phyA in vitro in a light-regulated manner. |
| At1g56430 | 0.498 | | Encodes a protein with nicotianamine synthase activity. Related to iron stress and homeostasis according to the publications in TAIR. |
| At2g18300 | 0.488 | | DNA-binding bHLH protein involved in positive regulation of cell elongation and proliferation and, negative control of plant immunity. |
| At5g61420 | 0.481 | | Encodes a nuclear localized member of the MYB transcription factor family. Involved in positive regulation of aliphatic glucosinolate biosynthesis.Expression is induced by touch, wounding and glucose. |
| At5g46270 | 0.481 | | Disease resistance protein (TIR-NBS-LRR class) |
| At2g23610 | 0.467 | | Encodes a protein shown to have carboxylesterase activity, methyl IAA esterase activity, and methyl jasmonate esterase activity in vitro. |
| At5g11610 | 0.459 | | Exostosin family protein, involved in protein glycosylation |
| At1g13110 | 0.459 | | Member of CYP71B The mRNA is cell-to-cell mobile. |
| At5g59080 | 0.454 | | Hypothetical protein, response to oxidative stress |
| At5g61590 | 0.454 | | Encodes an AP2/ERF-type transcription factor that is preferentially expressed in the epidermis and induced by darkness and negatively regulates cuticular wax biosynthesis. Provide resistance to shoot dehydration and low-temperature stress. |
| At5g44820 | 0.450 | | Nucleotide-diphospho-sugar transferase family protein |
| At3g47570 | 0.448 | | Leucine-rich repeat protein kinase family protein |
| At3g06070 | 0.442 | | Hypothetical protein |
| At3g56360 | 0.435 | | Hypothetical protein |
| At3g21080 | 0.434 | | ABC transporter-like protein |
| At2g41180 | 0.434 | | VQ motif-containing protein, function as activators of WRKY33 in plant defense against necrotrophic pathogens (TAIR publications) |
| At1g04240 | 0.433 | | Regulates multiple auxin responses in roots. It is induced rapidly by IAA, and has been shown to be phosphorylated by oat phytochrome A in vitro. |
| At5g02760 | 0.429 | | Encodes a phosphatase that functions in sustaining proper leaf longevity and preventing early senescence by suppressing or perturbing SARK-mediated senescence signal transduction. |
| At3g61210 | 0.425 | | S-adenosyl-L-methionine-dependent methyltransferases superfamily protein |
| At5g18240 | 0.422 | | Encodes MYR1, regulates flowering time, petiole elongation, and lateral shoot outgrowth in Arabidopsis (TAIR publications) |
| At5g02600 | 0.420 | | Encodes a phloem mobile metal binding protein necessary for phloem function and root meristem maintenance. |
| At5g58390 | 0.420 | | Peroxidase superfamily protein |

**TABLE S7** **Secondary-structure composition of each predicted TRQA1 model, determined using PROMOTIF (Hutchinson and Thornton, 1996).** For each model, the number of residues and the percentage of each secondary-structure type are shown.

| **At1g47400** | **β-strand -**  **number of residues (percentage)** | **α-helix -**  **number of residues (percentage)** | **3-10 helix -**  **number of residues (percentage)** | **Loop -**  **number of residues (percentage)** |
| --- | --- | --- | --- | --- |
| model1 | 0 (0.0%) | 12 (24.0%) | 3 (6.0%) | 35 (70.0%) |
| model2 | 0 (0.0%) | 9 (18.0%) | 0 (0.0%) | 41 (82.0%) |
| model3 | 0 (0.0%) | 12 (24.0%) | 0 (0.0%) | 38 (76.0%) |
| model4 | 0 (0.0%) | 12 (24.0%) | 0 (0.0%) | 38 (76.0%) |
| model5 | 0 (0.0%) | 9 (18.0%) | 0 (0.0%) | 41 (82.0%) |

**TABLE S8** **(File: Supplementary Tables.xlsx) Complete list of predicted transcription factor (TF)-binding sites in the 2-kb promoter region upstream of the *QQS* start codon.** Binding sites were identified using PlantRegMap (http://plantregmap.gao-lab.org/binding_site_prediction.php) (Tian et al., 2019) with the 2-kb region upstream of the *QQS* start codon as the query sequence (*P* ≤ 1 × 10^-4^).

**Table S9** **(File: Supplementary Tables.xlsx) Complete list of predicted *cis*-elements in the 2-kb promoter region upstream of the *QQS* start codon.** *Cis*-elements were identified using PlantCARE (http://bioinformatics.psb.ugent.be/webtools/plantcare/html/) (Lescot et al., 2002) and PLACE (https://www.dna.affrc.go.jp/PLACE/?action=newplace) (Higo et al., 1999), with the 2-kb region upstream of the *QQS* start codon used as the query sequence.

# References

Alam, I., Wu, X., Yu, Q., and Ge, L. (2022). Comprehensive Genomic Analysis of G2-like Transcription Factor Genes and Their Role in Development and Abiotic Stresses in Arabidopsis. *Diversity* 14**,** 228. doi: 10.3390/d14030228

Ambawat, S., Sharma, P., Yadav, N.R., and Yadav, R.C. (2013). MYB transcription factor genes as regulators for plant responses: an overview. *Physiol. Mol. Biol. Plants* 19**,** 307-321. doi: 10.1007/s12298-013-0179-1

Bakshi, M., and Oelmüller, R. (2014). WRKY transcription factors. *Plant Signal. Behav.* 9**,** e27700. doi: 10.4161/psb.27700

Camacho, C., Coulouris, G., Avagyan, V., Ma, N., Papadopoulos, J., Bealer, K., et al. (2009). BLAST+: architecture and applications. *BMC Bioinformatics* 10**,** 421. doi: 10.1186/1471-2105-10-421

Chandran, D., Rickert, J., Huang, Y., Steinwand, Michael A., Marr, Sharon K., and Wildermuth, Mary C. (2014). Atypical E2F transcriptional repressor DEL1 acts at the intersection of plant growth and immunity by controlling the hormone salicylic acid. *Cell Host Microbe* 15**,** 506-513. doi: 10.1016/j.chom.2014.03.007

Chen, C., Li, Y., Zhang, H., Ma, Q., Wei, Z., Chen, J., et al. (2021). Genome-Wide Analysis of the RAV Transcription Factor Genes in Rice Reveals Their Response Patterns to Hormones and Virus Infection. *Viruses* 13**,** 752. doi: 10.3390/v13050752

Cvitanich, C., Pallisgaard, N., Nielsen, K.A., Hansen, A.C., Larsen, K., Pihakaski-Maunsbach, K., et al. (2000). CPP1, a DNA-binding protein involved in the expression of a soybean leghemoglobin c3 gene. *Proc. Natl. Acad. Sci.* 97**,** 8163-8168. doi: 10.1073/pnas.090468497

Deng, Z., Yang, Z., Liu, X., Dai, X., Zhang, J., and Deng, K. (2023). Genome-Wide Identification and Expression Analysis of C3H Zinc Finger Family in Potato (Solanum tuberosum L.). *Int. J. Mol. Sci.* 24**,** 12888. doi: 10.3390/ijms241612888

Diao, P., Chen, C., Zhang, Y., Meng, Q., Lv, W., and Ma, N. (2020). The role of NAC transcription factor in plant cold response. *Plant Signal. Behav.* 15**,** 1785668. doi: 10.1080/15592324.2020.1785668

Doherty, C.J., Van Buskirk, H.A., Myers, S.J., and Thomashow, M.F. (2009). Roles for Arabidopsis CAMTA Transcription Factors in Cold-Regulated Gene Expression and Freezing Tolerance  *Plant Cell* 21**,** 972-984. doi: 10.1105/tpc.108.063958

Feurtado, J.A., Huang, D., Wicki-Stordeur, L., Hemstock, L.E., Potentier, M.S., Tsang, E.W.T., et al. (2011). The Arabidopsis C2H2 zinc finger INDETERMINATE DOMAIN1/ENHYDROUS promotes the transition to germination by regulating light and hormonal signaling during seed maturation. *Plant Cell* 23**,** 1772-1794. doi: 10.1105/tpc.111.085134

Gu, C., Guo, Z.-H., Hao, P.-P., Wang, G.-M., Jin, Z.-M., and Zhang, S.-L. (2017). Multiple regulatory roles of AP2/ERF transcription factor in angiosperm. *Bot. Stud.* 58**,** 6. doi: 10.1186/s40529-016-0159-1

Guan, P., Wang, R., Nacry, P., Breton, G., Kay, S.A., Pruneda-Paz, J.L., et al. (2014). Nitrate foraging by Arabidopsis roots is mediated by the transcription factor TCP20 through the systemic signaling pathway. *Proc. Natl. Acad. Sci.* 111**,** 15267-15272. doi: 10.1073/pnas.1411375111

Guo, J., Bai, X., Dai, K., Yuan, X., Guo, P., Zhou, M., et al. (2021a). Identification of GATA transcription factors in Brachypodium distachyon and functional characterization of BdGATA13 in drought tolerance and response to gibberellins. *Front. Plant Sci.* 12. doi: 10.3389/fpls.2021.763665

Guo, J., Sun, B., He, H., Zhang, Y., Tian, H., and Wang, B. (2021b). Current understanding of bHLH transcription factors in plant abiotic stress tolerance. *Int. J. Mol. Sci.* 22**,** 4921. doi: 10.3390/ijms22094921

Higo, K., Ugawa, Y., Iwamoto, M., and Korenaga, T. (1999). Plant cis-acting regulatory DNA elements (PLACE) database: 1999. *Nucleic Acids Res.* 27**,** 297-300. doi: 10.1093/nar/27.1.297

Hutchinson, E.G., and Thornton, J.M. (1996). PROMOTIF—a program to identify and analyze structural motifs in proteins. *Protein Sci.* 5**,** 212-220. doi: 10.1002/pro.5560050204

Jiang, D., Xia, M., Xing, H., Gong, M., Jiang, Y., Liu, H., et al. (2023). Exploring the Heat Shock Transcription Factor (HSF) Gene Family in Ginger: A Genome-Wide Investigation on Evolution, Expression Profiling, and Response to Developmental and Abiotic Stresses. *Plants* 12**,** 2999. doi: 10.3390/plants12162999

Jumper, J., Evans, R., Pritzel, A., Green, T., Figurnov, M., Ronneberger, O., et al. (2021). Highly accurate protein structure prediction with AlphaFold. *Nature* 596**,** 583-589. doi: 10.1038/s41586-021-03819-2

Karve, R., Suárez-Román, F., and Iyer-Pascuzzi, A.S. (2016). The Transcription Factor NIN-LIKE PROTEIN7 Controls Border-Like Cell Release  *Plant Physiol.* 171**,** 2101-2111. doi: 10.1104/pp.16.00453

Lescot, M., Déhais, P., Thijs, G., Marchal, K., Moreau, Y., Van de Peer, Y., et al. (2002). PlantCARE, a database of plant cis-acting regulatory elements and a portal to tools for in silico analysis of promoter sequences. *Nucleic Acids Res.* 30**,** 325-327. doi: 10.1093/nar/30.1.325

Li, M., Chen, H., Chen, J., Chang, M., Palmer, I.A., Gassmann, W., et al. (2018a). TCP transcription factors interact with NPR1 and contribute redundantly to systemic acquired resistance. *Front. Plant Sci.* 9. doi: 10.3389/fpls.2018.01153

Li, Q.-F., Lu, J., Yu, J.-W., Zhang, C.-Q., He, J.-X., and Liu, Q.-Q. (2018b). The brassinosteroid-regulated transcription factors BZR1/BES1 function as a coordinator in multisignal-regulated plant growth. *BBA Gene Regul. Mech.* 1861**,** 561-571. doi: 10.1016/j.bbagrm.2018.04.003

Li, S.-B., Xie, Z.-Z., Hu, C.-G., and Zhang, J.-Z. (2016). A review of auxin response factors (ARFs) in plants. *Front. Plant Sci.* 7. doi: 10.3389/fpls.2016.00047

Li, Y., Yang, Z., Zhang, Y., Guo, J., Liu, L., Wang, C., et al. (2022). The roles of HD-ZIP proteins in plant abiotic stress tolerance. *Front. Plant Sci.* 13. doi: 10.3389/fpls.2022.1027071

Liu, H., Tang, X., Zhang, N., Li, S., and Si, H. (2023). Role of bZIP Transcription Factors in Plant Salt Stress. *Int. J. Mol. Sci.* 24**,** 7893. doi: 10.3390/ijms24097893

Liu, K.-H., Liu, M., Lin, Z., Wang, Z.-F., Chen, B., Liu, C., et al. (2022a). NIN-like protein 7 transcription factor is a plant nitrate sensor. *Science* 377**,** 1419-1425. doi: 10.1126/science.add1104

Liu, Y., Khan, A.R., and Gan, Y. (2022b). C2H2 Zinc Finger Proteins Response to Abiotic Stress in Plants. *Int. J. Mol. Sci.* 23**,** 2730. doi: 10.3390/ijms23052730

Ma, B., Nian, L., Ain, N.U., Liu, X., Yang, Y., Zhu, X., et al. (2022). Genome-Wide Identification and Expression Profiling of the SRS Gene Family in Melilotus albus Reveals Functions in Various Stress Conditions. *Plants* 11**,** 3101. doi: 10.3390/plants11223101

Meng, E.C., Goddard, T.D., Pettersen, E.F., Couch, G.S., Pearson, Z.J., Morris, J.H., et al. (2023). UCSF ChimeraX: tools for structure building and analysis. *Protein Sci.* 32**,** e4792. doi: 10.1002/pro.4792

Mirdita, M., Schütze, K., Moriwaki, Y., Heo, L., Ovchinnikov, S., and Steinegger, M. (2022). ColabFold: making protein folding accessible to all. *Nat. Methods* 19**,** 679-682. doi: 10.1038/s41592-022-01488-1

Mou, Y., Yuan, C., Sun, Q., Yan, C., Zhao, X., Wang, J., et al. (2022). MIKC-type MADS-box transcription factor gene family in peanut: Genome-wide characterization and expression analysis under abiotic stress. *Front. Plant Sci.* 13. doi: 10.3389/fpls.2022.980933

Nakashima, K., Takasaki, H., Mizoi, J., Shinozaki, K., and Yamaguchi-Shinozaki, K. (2012). NAC transcription factors in plant abiotic stress responses. *BBA Gene Regul. Mech.* 1819**,** 97-103. doi: 10.1016/j.bbagrm.2011.10.005

Nan, H., Lin, Y., Wang, X., and Gao, L. (2021). Comprehensive genomic analysis and expression profiling of cysteine-rich polycomb-like transcription factor gene family in tea tree. *Hortic. Plant J.* 7**,** 469-478. doi: 10.1016/j.hpj.2021.03.001

Reiser, L., Bakker, E., Subramaniam, S., Chen, X., Sawant, S., Khosa, K., et al. (2024). The Arabidopsis information resource in 2024. *Genetics***,** iyae027. doi: 10.1093/genetics/iyae027

Roy, A., Kucukural, A., and Zhang, Y. (2010). I-TASSER: a unified platform for automated protein structure and function prediction. *Nat. Protoc.* 5**,** 725-738. doi: 10.1038/nprot.2010.5

Sahu, A., Singh, R., and Verma, P.K. (2023). Plant BBR/BPC transcription factors: unlocking multilayered regulation in development, stress and immunity. *Planta* 258**,** 31. doi: 10.1007/s00425-023-04188-y

Schmid, M., Davison, T.S., Henz, S.R., Pape, U.J., Demar, M., Vingron, M., et al. (2005). A gene expression map of Arabidopsis thaliana development. *Nat. Genet.* 37**,** 501-506. doi: 10.1038/ng1543

Sidhu, N.S., Pruthi, G., Singh, S., Bishnoi, R., and Singla, D. (2020). Genome-wide identification and analysis of GRAS transcription factors in the bottle gourd genome. *Sci. Rep.* 10**,** 14338. doi: 10.1038/s41598-020-71240-2

Suzuki, M., and McCarty, D.R. (2008). Functional symmetry of the B3 network controlling seed development. *Curr. Opin. Plant Biol.* 11**,** 548-553. doi: 10.1016/j.pbi.2008.06.015

Szklarczyk, D., Gable, A.L., Lyon, D., Junge, A., Wyder, S., Huerta-Cepas, J., et al. (2019). STRING v11: protein–protein association networks with increased coverage, supporting functional discovery in genome-wide experimental datasets. *Nucleic Acids Res.* 47**,** D607-D613. doi: 10.1093/nar/gky1131

Tian, F., Yang, D.-C., Meng, Y.-Q., Jin, J., and Gao, G. (2019). PlantRegMap: charting functional regulatory maps in plants. *Nucleic Acids Res.* 48**,** D1104-D1113. doi: 10.1093/nar/gkz1020

Toufighi, K., Brady, S.M., Austin, R., Ly, E., and Provart, N.J. (2005). The Botany Array Resource: e‐Northerns, expression angling, and promoter analyses. *Plant J.* 43**,** 153-163. doi: 10.1111/j.1365-313X.2005.02437.x

Varadi, M., Anyango, S., Deshpande, M., Nair, S., Natassia, C., Yordanova, G., et al. (2021). AlphaFold Protein Structure Database: massively expanding the structural coverage of protein-sequence space with high-accuracy models. *Nucleic Acids Res.* 50**,** D439-D444. doi: 10.1093/nar/gkab1061

Verma, S., and Bhatia, S. (2019). A comprehensive analysis of the B3 superfamily identifies tissue-specific and stress-responsive genes in chickpea (Cicer arietinum L.). *3 Biotech* 9**,** 346. doi: 10.1007/s13205-019-1875-5

Wang, F., Lin, R., Feng, J., Qiu, D., Chen, W., and Xu, S. (2015). Wheat bHLH transcription factor gene, TabHLH060, enhances susceptibility of transgenic Arabidopsis thaliana to Pseudomonas syringae. *Physiol. Mol. Plant Pathol.* 90**,** 123-130. doi: 10.1016/j.pmpp.2015.04.007

Wang, Z., Zhang, R., Cheng, Y., Lei, P., Song, W., Zheng, W., et al. (2021). Genome-wide identification, evolution, and expression analysis of LBD transcription factor family in bread wheat (Triticum aestivum L.). *Front. Plant Sci.* 12. doi: 10.3389/fpls.2021.721253

Winter, D., Vinegar, B., Nahal, H., Ammar, R., Wilson, G.V., and Provart, N.J. (2007). An “Electronic Fluorescent Pictograph” browser for exploring and analyzing large-scale biological data sets. *PLOS ONE* 2**,** e718. doi: 10.1371/journal.pone.0000718

Wu, Y., Li, X., Zhang, J., Zhao, H., Tan, S., Xu, W., et al. (2022). ERF subfamily transcription factors and their function in plant responses to abiotic stresses. *Front. Plant Sci.* 13. doi: 10.3389/fpls.2022.1042084

Xiao, P., Feng, J.-W., Zhu, X.-T., and Gao, J. (2021). Evolution analyses of CAMTA transcription factor in plants and its enhancing effect on cold-tolerance. *Front. Plant Sci.* 12. doi: 10.3389/fpls.2021.758187

Yang, J., Tang, Z., Yang, W., Huang, Q., Wang, Y., Huang, M., et al. (2023). Genome-wide characterization and identification of Trihelix transcription factors and expression profiling in response to abiotic stresses in Chinese Willow (Salix matsudana Koidz). *Front. Plant Sci.* 14. doi: 10.3389/fpls.2023.1125519

Zou, X., and Sun, H. (2023). DOF transcription factors: specific regulators of plant biological processes. *Front. Plant Sci.* 14. doi: 10.3389/fpls.2023.1044918
